# Seamless Transition to Post-Quantum TLS 1.3: A Hybrid Approach Using Identity-Based Encryption

**DOI:** 10.3390/s24227300

**Published:** 2024-11-15

**Authors:** Thiago Leucz Astrizi, Ricardo Custódio

**Affiliations:** Graduate Program on Computer Science, Department of Informatics and Statistics, Federal University of Santa Catarina (UFSC), Florianópolis 88040-370, SC, Brazil; ricardo.custodio@ufsc.br

**Keywords:** hybrid post-quantum cryptography, KEMTLS, network security

## Abstract

We propose a novel solution to streamline the migration of existing Transport Layer Security (TLS) protocol implementations to a post-quantum Key Encapsulation Mechanism for Transport Layer Security (KEMTLS). By leveraging Identity-Based Encryption (IBE), our solution minimizes the necessary modifications to the surrounding infrastructure, enabling the reuse of existing keys and certificates. We provide a proof-of-concept implementation and performance analysis, demonstrating the practical feasibility and effectiveness of our proposed approach.

## 1. Introduction

Transport Layer Security (TLS) is a widely used cryptographic protocol that ensures secure communication over computer networks. It plays a vital role in protecting web pages and enabling secure machine-to-machine interactions. TLS 1.3, specified in RFC 8446 [1], enables secure message exchange over untrusted networks by establishing a shared secret between communicating parties. Unlike protocols that rely solely on symmetric encryption, TLS combines multiple cryptographic techniques, including the following.

Diffie-Hellman Key Agreement Protocol:

TLS employs the Diffie–Hellman Key Agreement Protocol, allowing the client and server to establish a shared secret without directly transmitting it. By leveraging asymmetric cryptography, both parties can independently compute the shared secret from public components.

TLS uses digital signatures to authenticate the server and, optionally, the client. This authentication process is based on public-key cryptography, which relies on a pair of public and private keys. Certification Authorities (CAs) issue digital certificates to validate the authenticity of the public keys, establishing trust between the communicating parties.

**Figure 1:**
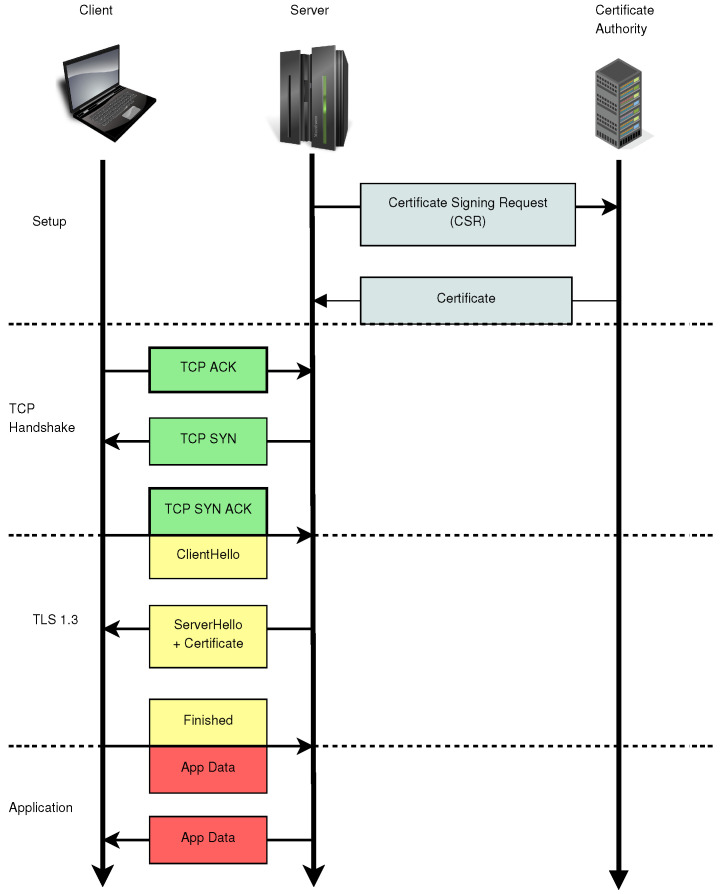
Diagram illustrating the usage of TLS 1.3. The server must first obtain a certificate from a Certificate Authority before initiating the protocol issuing a Certificate Signing Request (CSR) to authenticate itself, presenting the public key to be certificated and proving that it has the corresponding secret key (proof of knowledge). The Transmission Control Protocol (TCP) handshake, typically managed by the operating system, establishes the connection between the client and server. TLS 1.3 then secures the connection, enabling secret sharing between the client and server, which allows encryption of application data.

Once established, the shared secret allows TLS to use symmetric encryption for efficient bulk data transfer, ensuring both confidentiality and integrity. This combination of asymmetric and symmetric cryptography provides TLS with a robust and efficient security foundation. A diagram illustrating how TLS 1.3 establishes secure connections is shown in Figure 1.

Cryptographically Relevant Quantum Computers (CRQCs) pose a significant threat to TLS 1.3. With their ability to efficiently execute Shor’s algorithm, CRQCs could factor large integers and solve discrete logarithms exponentially faster than classical computers, compromising current public-key cryptographic algorithms [2]. This capability compromises the security of current public-key cryptographic algorithms, including RSA signature (named by authors Rivest, Shamir, and Adleman) and Elliptic Curve Cryptography (ECC). Recognizing this looming threat, the National Institute of Standards and Technology (NIST) initiated a post-quantum cryptography standardization program in 2016 [3] to identify algorithms resistant to quantum attacks. By 2022, NIST selected several post-quantum algorithms for further testing and implementation, paving the way for secure communication in the post-quantum era.

Adapting TLS 1.3 to resist quantum attacks typically involves replacing the Diffie–Hellman key agreement with a post-quantum Key Encapsulation Mechanism (KEM) and substituting classical signatures with post-quantum alternatives. These modifications often increase message sizes due to the larger post-quantum keys and signatures [4,5]. One solution to address this issue is the Key Encapsulation Mechanism for the Transport Layer Security (KEMTLS) protocol, developed by Peter Schwabe, Douglas Stebila, and Thom Wiggers [6]. KEMTLS leverages post-quantum key encapsulation mechanisms (KEMs) for both key exchange and authentication, which reduces message sizes compared to traditional TLS handshakes and mitigates the performance penalties typically associated with post-quantum cryptography. However, this approach introduces an additional round trip for secret sharing, which may affect latency. When comparing KEMTLS to TLS 1.3 using post-quantum algorithms, KEMTLS generally requires less memory and exhibits slightly better performance timings [7,8].

The transition to post-quantum technology is expected to be gradual, with hybrid approaches that combine classical and post-quantum algorithms to achieve security levels equivalent to the strongest component. This strategy mitigates the risks associated with the early stages of post-quantum algorithms, which require thorough validation. The recent compromise of SIKE, despite its progress in the NIST standardization process, highlights the need for caution and ongoing evaluation [9,10]. The Open Quantum Safe project strongly recommends the use of hybrid algorithms during the migration process [11], and NIST recognizes hybrid constructions as compatible with its standards [12]. More recently, draft standards proposing the use of hybrid algorithms in TLS have emerged, such as in [13].

The TLS ecosystem extends beyond the protocol itself, encompassing certification authorities (CAs), certificate validation software, revocation status checking, trust in root CAs, and updates to both client and server applications. Transitioning to post-quantum technologies requires significant updates across this entire ecosystem, including changes to CAs, Online Certificate Status Protocol (OCSP) responders, and Certificate Revocation Lists (CRLs). Several factors could impede this transition. CAs must support both classical and post-quantum algorithms to issue and validate certificates during the transition period. Additionally, generating new keys and certificates for millions of TLS servers is a complex and time-consuming process. While large organizations may manage this transition more efficiently, smaller entities and resource-constrained Internet-of-Things (IoT) devices will likely face significant challenges and delays.

To address these challenges, we propose a solution that enables post-quantum security for TLS communications without requiring immediate support for post-quantum algorithms from CAs or servers. Our approach allows systems to adopt post-quantum TLS while retaining existing certificates and keys, thereby streamlining the transition process. Compared to other proposals such as KEMTLS, our solution minimizes the necessary modifications to the ecosystem, enabling broader and faster adoption.

Previous works addressing migration challenges, such as [14], highlight the difficulty of regenerating certificate chains to authenticate post-quantum keys. That paper proposes a gradual migration method where the root certificate can initially remain unchanged, using a hash-based signature to allow different signature algorithms within the same certificate chain. However, this approach still requires users to generate new keys and create updated versions of other certificates in the chain.

Our Proposal

We develop a hybrid KEMTLS variant that combines post-quantum and classical key encapsulation mechanisms. The classical component leverages the existing public key infrastructure, while the post-quantum component utilizes identity-based encryption (IBE), eliminating the need for post-quantum keys in TLS certificates. This novel combination allows existing certificates to be used for post-quantum communications, preserving current certificate generation and authentication processes. Additionally, current Elliptic Curve Digital Signature Algorithm (ECDSA)-based leaf certificates can be used for both message signing in TLS 1.3 and message encapsulation within our proposed protocol. We demonstrate in a new theorem that using the same key for both ECDSA signatures and a specific Diffie–Hellman-based KEM does not compromise security, following established ECDSA security models.

In identity-based systems, a server’s domain name or another identifier serves as its public key, eliminating the need to transmit or certify public keys as they are inherently public. However, secret keys for individual identities are generated by a Private Key Generator (PKG) and securely delivered to the corresponding servers.

Our proposal complements and can be used alongside the migration strategy from [14]. While their strategy addresses the challenge of updating root certificates, our approach tackles the difficulty of regenerating keys and certificate chains, making it suitable for an earlier phase of the migration process. According to the taxonomy in [15], we offer a new hybrid post-quantum key exchange solution, as our method focuses specifically on the key exchange aspect of the TLS protocol, deferring the authentication component to a later stage in the migration.

Given our approach, one might question the necessity of maintaining a traditional Public Key Infrastructure (PKI). While a fully hybrid scheme using IBE for both components is theoretically possible—eliminating the need for certificates and certificate authorities—there are practical challenges for general TLS usage. Specifically, agreeing on the PKG during initial communication presents difficulties. Existing IBE-based post-quantum TLS proposals often focus on constrained environments with predetermined PKGs, such as IoT devices (e.g., Scott, 2023 [16] and Ducas, 2014 [17]). Our approach aims for broader applicability by incorporating PKG information within TLS extensions, either through certificates or the protocol itself, while leveraging existing infrastructure for the classical component of the hybrid scheme.

### 1.1. Addressing IBE Challenges in TLS

Our proposal addresses two key challenges of incorporating IBE into TLS.

Revocation Problem

The problem consists in using a server’s domain name as its identity introduces a revocation challenge. Revoking the server’s secret key effectively revokes the entire domain, which is impractical. Common solutions include appending timestamps or additional strings to the identity, but these often require additional certification steps. Our solution integrates the classical public key from the certificate into the identity, aligning it with existing revocation mechanisms and making the process more manageable.

Key Escrow Problem

IBE’s reliance on a PKG to generate secret keys for all parties poses a key escrow risk, particularly in a widely deployed protocol like TLS. Our hybrid approach mitigates this by combining IBE with a traditional public key algorithm. By using combiners, we ensure that the security of the scheme is at least as strong as the most robust component. Even if the PKG is compromised, the system is not entirely compromised, as the key encapsulation mechanism remains secure provided the classical public key algorithm remains secure. In the worst-case scenario—where a server key is leaked—the protocol reverts to a classical Diffie–Hellman-based system, maintaining TLS 1.3 security levels but losing post-quantum resistance. This trade-off is acceptable as a transitional solution, enabling a smoother migration to full post-quantum security. As the ecosystem evolves, the IBE component may be replaced by more conventional post-quantum PKI mechanisms.

### 1.2. Overview of Our Contribution

Our main contribution is the development of a method that facilitates the transition to a post-quantum TLS with minimal infrastructure changes, enabling a faster and more seamless migration. This approach leverages a hybrid KEM scheme that integrates existing PKI with IBE. A key innovation of our proposal lies in its capacity to reuse current certificates for the classical portion while delegating post-quantum operations to an IBE mechanism.

**Figure 2:**
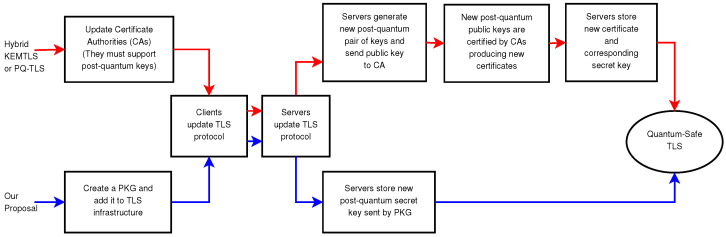
This diagram compares the necessary steps to migrate from TLS 1.3 to a typical KEMTLS or PQ-TLS proposal (the red arrows) with the steps necessary to migrate from TLS 1.3 to our proposal (blue arrows).

This dual-layered approach ensures compatibility with current PKI standards, reducing the complexity of migration steps and eliminating the immediate need for updates from CAs or server infrastructure. By maintaining the use of classical certificates, our solution can automate parts of the migration process, easing the burden on server administrators as detailed in Section 3.4. The comparative simplicity of our proposal is illustrated in Figure 2, which provides a step-by-step diagram showcasing how our approach contrasts with KEMTLS and highlights its reduced complexity.

Our methodology also addresses significant theoretical challenges. To our knowledge, the combination of PKI-based schemes with IBE via combiners, as proposed here, has not been explored previously. This combination allows existing keys to be repurposed without compromising security, a novelty in itself. To substantiate these claims, we include rigorous security models and proofs in the appendices, ensuring that the hybrid approach withstands both classical and quantum threats.

Empirical comparisons and performance analyses are provided in Section 4, where we evaluate our proposal against other post-quantum and hybrid KEMTLS implementations. Despite the larger message sizes, the results demonstrate that our timings remain competitive, validating the approach as a viable step toward a fully quantum-safe TLS.

### 1.3. Paper Content

This paper is organized as follows:

- Section 2 introduces the notation used throughout the paper and describes the cryptographic algorithms central to our proposal and the KEMTLS protocol. It provides a detailed breakdown of KEMs, IBE, and their integration within the TLS protocol.
- Section 3 presents our novel integration of post-quantum identity-based encryption algorithms with classical public key algorithms. We develop a security model for the resulting hybrid cryptographic primitive and discuss its practical and theoretical security implications.
- Section 4 compares the performance of our hybrid KEMTLS protocol with the current TLS 1.3 implementation and other hybrid approaches. Performance metrics are evaluated in both simulated network environments and real-world Internet configurations.
- Section 5 concludes by summarizing our findings and discussing potential directions for future research in post-quantum cryptography in network security protocols. This section reflects on the broader implications for cybersecurity and the transition to quantum-resistant cryptographic systems.
- Appendix A contains the full security proof supporting our claims about the hybrid KEMTLS’s resilience against classical and quantum cryptographic attacks as outlined in Theorem 1.
- Appendix B provides the complete security proof of Theorem 2, substantiating our claim that the same elliptic curve keys can be used interchangeably for both our proposed KEM algorithm and ECDSA signatures without compromising security.

## 2. Preliminaries

### 2.1. Notation

We use uppercase letters to represent cryptographic schemes (e.g., KEM for key encapsulation mechanisms and SIG for signature schemes). Each algorithm in these schemes is prefixed by the scheme name for easy identification. For example, KEM.KeyGen and SIG.KeyGen refer to different key generation algorithms, even though they share the same name after the prefix. Algorithms not associated with a specific cryptographic scheme are denoted without a prefix, with the first letter capitalized.

Sets are represented by single uppercase letters, such as *A* and *M*, while generic set elements are denoted by lowercase names like msg and *k*. The symbol λ is used to denote security parameters in cryptographic schemes.

When describing algorithms or attack games, the symbol “←” denotes an assignment. We also use “$\;\overset{\;\$}{\leftarrow }\;$” to indicate that an element is sampled uniformly at random from the set on the right side of the operator and assigned to the variable on the left. If a returned value is irrelevant and should be discarded, it is assigned to the variable “–”.

In attack games, we use A to represent an adversary attempting to break the security of a cryptographic scheme. The adversary is modeled as a tuple of algorithms, depending on the rules of the specific attack game. Subscripts such as $A_{1}$ and $A_{2}$ are used to distinguish between the different algorithms that make up A and to differentiate stages of the attack game when A is invoked. If A is able to query oracles during the game, the oracles are represented as superscripts over A.

The notation Pr[A] denotes the probability of a particular event *A* occurring. And Adv(A) is the probability of a given adversary A breaking the security of a specific cryptographic primitive, with subscripts indicating which primitive is being broken and superscripts indicating the security definition.

### 2.2. Key Encapsulation Mechanisms (KEMs)

A Key Encapsulation Mechanism (KEM) is defined by the following algorithms:

- Key Generation: $KEM.KeyGen\left(1^{\lambda }\right)\to \left(pk,sk\right)$: Given the security parameter $1^{\lambda }$, this probabilistic polynomially bounded algorithm generates a secret key sk and a public key pk.
- Encapsulation: KEM.Encaps(pk)→(k,c): Given a public key pk, this probabilistic polynomially bounded algorithm generates a secret k∈K and a ciphertext c∈C, where *K* is the key space and *C* the ciphertext space.
- Decapsulation: KEM.Decaps(sk,c)→k: Given a secret key sk and a ciphertext c∈C, this deterministic polynomially bounded algorithm recovers the corresponding secret k∈K.

The security of a public-key KEM is evaluated through two attack games, referred to as Game 0 and Game 1, detailed below as Algorithms 1 and 2. For any adversary A, we let $W_{0}$ represent the event where A returns one in Attack Game 0 and $W_{1}$ represent the event where A returns one in Attack Game 1. The advantage of adversary A is defined as $Adv_{KEM}^{IND-CCA}\left(A\right)=\left|Pr\left[W_{0}\right]-Pr\left[W_{1}\right]\right|$. A KEM is considered secure if this advantage is negligible for all adversaries A.
**Algorithm 1** KEM Attack Game 01:$\left(sk,pk\right)\leftarrow \mathrm{KEM}.\mathrm{KeyGen}\left(1^{\lambda }\right)$2:$\left(c^{*},k_{0}\right)\leftarrow \mathrm{KEM}.\mathrm{Encaps}\left(pk\right)$3:$b\leftarrow A^{\mathrm{KEM}.\mathrm{Decaps}(sk,\cdot )}\left(pk,c^{*},k_{0}\right)$  ▹ Run adversary A, allowing decapsulation queries.4:            ▹ The adversary controls the ciphertext to be decapsulated.5:            ▹ The secret key sk remains hidden and cannot be accessed.6:**return** *b*

**Algorithm 2** KEM Attack Game 1
1:

$\left(sk,pk\right)\leftarrow \mathrm{KEM}.\mathrm{KeyGen}\left(1^{\lambda }\right)$

2:

$\left(c^{*},-\right)\leftarrow \mathrm{KEM}.\mathrm{Encaps}\left(pk\right)$

3:$k_{1}\;\overset{\;\$}{\leftarrow }\;K$              ▹ Randomly chosen key from key space *K*4:

$b\leftarrow A^{\mathrm{KEM}.\mathrm{Decaps}(sk,\cdot )}\left(pk,c^{*},k_{1}\right)$

5:**return** *b*

KEM Attack Games

The adversary’s objective is to distinguish between two scenarios, and the advantage measures its ability to do so, thereby compromising the KEM’s security based on the indistinguishability under chosen-ciphertext attacks (IND-CCA).

In both games, the adversary is allowed to query an oracle for decapsulation using the secret key sk, but cannot request the decapsulation of the specific ciphertext $c^{*}$.

When KEMs are used for key exchange, users typically generate their key pairs independently of their identity. A certificate signed by a trusted authority and containing both the public key pk and the identity of its owner is then used to verify that the claimed identity matches the provided public key.

#### Diffie–Hellman KEM

A common Key Encapsulation Mechanism (KEM) is the Diffie–Hellman KEM (DHKEM), which operates over a multiplicative group *G* with generator *g* and order *q*, typically derived from an elliptic curve. The security of DHKEM relies on the computational difficulty of the Diffie–Hellman problem: given *g*, $g^{a}$, and $g^{b}$, we compute $g^{ab}$. This KEM uses a hash function $H:{\left\{0,1\right\}}^{*}\to K$, where *K* is the secret space, and assumes efficient algorithms exist to verify solutions for the Diffie–Hellman problem.

The secret key is a random exponent *x* such that 0<x<q, and the corresponding public key is $y=g^{x}$. The encapsulation and decapsulation algorithms are provided in Algorithms 3 and 4.

This KEM is described in further detail and has a proposed standardization in Section 3.1 of RFC 9180 [18].
**Algorithm 3** DHKEM.Encaps(*y*)1:**Input:** Public key *y*2:**Output:** Ciphertext *c*, Shared secret *k*3:$x^{\prime }\;\overset{\;\$}{\leftarrow }\;Z_{q}$4:$y^{\prime }\leftarrow g^{x^{\prime }}$5:$s\leftarrow y^{x^{\prime }}$6:$k\leftarrow \mathrm{Hash}(s\|y^{\prime }\|y)$7:$c\leftarrow y^{\prime }$8:**return**(c,k)

**Algorithm 4** DHKEM.Decaps(*x*, *c*)
1:**Input:** Secret key *x*, Ciphertext *c*2:**Output:** Shared secret *k*3:

$y^{\prime }\leftarrow c$

4:

$s\leftarrow y^{\prime x}$

5:**return** $\mathrm{Hash}(s\|y^{\prime }\|y)$


### 2.3. Identity Based KEMs

An identity-based Key Encapsulation Mechanism (IDKEM) consists of the following algorithms:

- Key Generation: $IDKEM.KeyGen\left(1^{\lambda }\right)\to \left(mpk,msk\right)$: Given the security parameter $1^{\lambda }$, this probabilistic polynomially bounded algorithm generates a master public key mpk and a master secret key msk;
- Key Extraction: $IDKEM.Extract\left(msk,id\right)\to sk_{id}$: Given the master secret key msk and a string id, this probabilistic polynomially bounded algorithm generates a secret key $sk_{id}$ associated with identity id;
- Encapsulation: IDKEM.Encaps(mpk,id)→(k,c): Given the master public key mpk and an identity string id, this probabilistic polynomially bounded algorithm produces a secret *k*, and a ciphertext *c*;
- Decapsulation: $IDKEM.Decaps(sk_{id},c)\to k$: Given a secret key $sk_{id}$ associated with identity id and a ciphertext *c*, this deterministic polynomially bounded algorithm recovers the secret *k*.

The security of an IDKEM is evaluated through attack games similar to those used for traditional KEMs. These games are depicted in IDKEM Attack Games 0 and 1 (Algorithms 5 and 6) as well as in Algorithm 7.
**Algorithm 5** IDKEM Attack Game 01:$\left(msk,mpk\right)\leftarrow \mathrm{IDKEM}.\mathrm{KeyGen}\left(1^{\lambda }\right)$2:$\left(id^{*},st\right)\leftarrow A_{1}^{\mathrm{Decaps}(msk,\cdot ),\mathrm{IDKEM}.\mathrm{Extract}(msk,\cdot )}\left(mpk\right)$3:$\left(c^{*},k_{0}\right)\leftarrow \mathrm{IDKEM}.\mathrm{Encaps}\left(mpk,id^{*}\right)$4:$b\leftarrow A_{2}^{\mathrm{Decaps}(msk,\cdot ),\mathrm{IDKEM}.\mathrm{Extract}(msk,\cdot )}\left(st,c^{*},k_{0}\right)$    ▹ Queries to Decaps are defined in Algorithm 7.5:**return** *b*

The adversary’s objective is to distinguish between the two scenarios presented in the attack games. The adversary’s advantage, denoted as $Adv_{IDKEM}^{IND-CCA}\left(A\right)$, measures their ability to differentiate between the games. It is defined as $|\mathrm{Pr}\left[W_{0}\right]-\mathrm{Pr}\left[W_{1}\right]|$, where $W_{0}$ and $W_{1}$ represent the events where the adversary outputs one in Attack Game 0 and Attack Game 1, respectively. A secure IDKEM ensures that this advantage is negligible for any adversary A.
**Algorithm 6** IDKEM Attack Game 11:$\left(msk,mpk\right)\leftarrow \mathrm{IDKEM}.\mathrm{KeyGen}\left(1^{\lambda }\right)$2:$\left(id^{*},st\right)\leftarrow A_{1}^{\mathrm{Decaps}(msk,\cdot ),\mathrm{IDKEM}.\mathrm{Extract}(msk,\cdot )}\left(mpk\right)$3:$\left(c^{*},-\right)\leftarrow \mathrm{IDKEM}.\mathrm{Encaps}\left(mpk,id^{*}\right)$4:$k_{1}\;\overset{\;\$}{\leftarrow }\;K$              ▹ Randomly chosen key from key space *K*5:$b\leftarrow A_{2}^{\mathrm{Decaps}(msk,\cdot ),\mathrm{IDKEM}.\mathrm{Extract}(msk,\cdot )}\left(st,c^{*},k_{1}\right)$6:**return** *b*

**Algorithm 7** Decapsulation Query for IDKEM Attack Games
1:Decaps(msk,id,c):2: $sk_{ID}\leftarrow \mathrm{IDKEM}.\mathrm{Extract}\left(msk,id\right)$3: **return** $\mathrm{IDKEM}.\mathrm{Decaps}(sk_{id},c)$


In the context of identity-based KEM attack games, the adversary can select the identity it wants to target. Initially, the adversary is given the master public key (mpk) and is allowed to send decapsulation queries to an oracle, as defined in Algorithm 7. Additionally, the adversary can query the oracle for the output of IDKEM.Extract, obtaining the secret key for any identity. After gathering this information, the adversary selects a target identity $id^{*}$. The adversary then receives a ciphertext associated with the chosen identity and either the secret encapsulated in the ciphertext or an unrelated secret, chosen uniformly at random. The adversary’s goal is to distinguish between these two scenarios. An example of identity-based KEM used in this article is the DLP-IBE proposed in [17] by Ducas, Lyubashevsky, and Prest.

### 2.4. Message Authentication Codes (MACs)

A Message Authentication Code (MAC) ensures the integrity and authenticity of a message. Defined over the sets (K,M,T), a MAC comsists of the following algorithms:

- Key Generation: $MAC.KeyGen(1^{\lambda })\to key$: Given the security parameter $1^{\lambda }$, this algorithm generates key∈K.
- Signing: MAC.Sign(key,msg)→tag: Given inputs key∈K and msg∈M, this algorithm generates tag∈T.
- Verifying signature: MAC.Verify(key,msg,tag)→b: Given a key key∈K, a message msg∈M, and a tag tag∈T, this algorithm outputs a value *b*, indicating either accept or reject.

A tag generated by MAC.Sign for a message is always accepted when verified with the same message and key.

For our hybrid construction, we assume that a MAC is secure if no adversary can forge an accepted tag for a chosen message, even when allowed to manipulate half of the bits of the key *k*, query a single signature, and perform a polynomial number of verification queries, manipulating and changing half of the key bits they control. The advantage of an adversary *A* is the probability of forging an accepted tag in the attack game illustrated below (Algorithms 8 and 9).
**Algorithm 8** MAC Attack Game1:$k\leftarrow \mathrm{MAC}.\mathrm{KeyGen}(1^{\lambda })$2:$k_{1}\|k_{2}\leftarrow k$3:$\left(msg^{*},b,k_{b},st\right)\leftarrow A_{1}\left(1^{\lambda }\right)$   ▹ First execution of adversary. Its state can be stored in st.4:**if** b=0 **then**5:    $k^{*}\leftarrow k_{b}\|k_{2}$6:**else**7:    $k^{*}\leftarrow k_{1}\|k_{b}$8:**end if**9:$tag^{*}\leftarrow \mathrm{MAC}.\mathrm{Sign}\left(k^{*},msg^{*}\right)$10:$\left(msg^{\prime },tag^{\prime }\right)\leftarrow A_{2}^{\mathrm{Verify}(\cdot ,\cdot ,\cdot )}\left(st,tag^{*}\right)$   ▹ Now adversary tries to produce a forgery.11:**return** $\mathrm{MAC}.\mathrm{Verify}(k^{*},msg^{\prime },tag^{\prime })$

**Algorithm 9** Verification Query for MAC Attack Game
1:Verify($k_{b},msg,tag$):2:**if** b=0 **then**3:    $k^{\prime }\leftarrow k_{b}\|k_{2}$4:
**else**
5:    $k^{\prime }\leftarrow k_{1}\|k_{b}$6:
**end if**
7:**return** $\mathrm{MAC}.\mathrm{Verify}(k^{\prime },msg,tag)$


In the MAC attack game, the adversary submits a message in a query and selects half of the bits of the MAC key. In response, the adversary receives the computed tag for the queried message. With this information and the ability to verify MAC tags, the adversary’s goal is to produce a forgery—a valid tag for a new message. The adversary’s advantage is the probability of successfully generating such a valid tag.

### 2.5. Signature Schemes

A signature scheme is defined by the following algorithms, operating over the sets (*M*, *S*) where *M* is the set of all possible messages and *S* is the set of signatures.

- Key Generation: $SIG.KeyGen\left(1^{\lambda }\right)\to \left(pk,sk\right)$: A probabilistic algorithm that, given a security parameter $1^{\lambda }$, generates a cryptographic key pair (pk,sk), where pk is the public key and sk is the secret key;
- Signing: SIG.Sign(sk,msg)→sig: A probabilistic algorithm that, given a secret key sk and a message msg∈M, produces a signature sig∈S;
- Signature Verification: SIG.Verify(pk,msg,sig)→b: A deterministic algorithm that, given a public key pk, a message msg∈M, and a signature sig∈S, returns a binary value *b*. If b=1, the signature is valid (accept); otherwise, it is invalid (reject).

A signature generated by Sign for a given message is always accepted when verified with the same message and key.

The security of a signature scheme is evaluated under the Unforgeability under Chosen Message Attacks (UF-CMA) model, formalized by Goldwasser et al. [19]. In this model, the adversary’s goal is to produce a forged signature. The adversary can adaptively request signatures for any message, but the forged signature must be for a new message, one that has not been previously queried. The adversary’s advantage is the probability of successfully returning one in the attack game described below in Algorithms 10 and 11.
**Algorithm 10** Signature Attack Game1:$\left(pk,sk\right)\leftarrow \mathrm{SIG}.\mathrm{KeyGen}\left(1^{\lambda }\right)$2:Q←∅3:$\left(msg^{*},sig^{*}\right)\leftarrow A^{SigningQuery(sk,\cdot )}\left(1^{\lambda },pk\right)$4:**if** $SIG.Verify(pk,msg^{*},sig^{*})=\mathrm{accept}$ **and** $msg^{*}\notin Q$ **then**5:    **return** 16:**else**7:    **return** 08:**end if**

**Algorithm 11** Signing Query for Sigature Attack Game
1:**procedure** SigningQuery(sk,msg):2:    Q←Q∪{msg}3:    **return** SIG.Sign(sk,msg)4:
**end procedure**



A signature scheme is considered secure under the UF-CMA model if, for any adversary A, its advantage is always a negligible function of the security parameter λ. This probability is denoted as $Adv_{SIG}^{UF-CMA}\left(A\right)$.

#### ECDSA Signature Scheme

An example of a signature scheme is the ECDSA, which operates over a group *G* derived from an elliptic curve. We treat *G* as a multiplicative group with order *q*, and *g* as one of its generators. ECDSA signatures are defined over $Z_{q}^{2}$, where *q* is the order of the elliptic curve. A hash function $Hash:M\to Z_{q}$ is used, where *M* represents the message space. The signature scheme is described in Algorithms 12 and 13.
**Algorithm 12** 
ECDSA.Sign(sk,msg)→(r,s)
1:x←sk2:**repeat**3:    $k\;\overset{\;\$}{\leftarrow }\;Z_{q}$4:    $y^{\prime }\leftarrow g^{k}$5:    We let *r* be the *x*-coordinate of the point $y^{\prime }$ on the elliptic curve *G* modulo *q*6:    $s\leftarrow \left(\mathrm{Hash}(msg)+rx\right)k^{-1}\;\left(\mathrm{mod}\;q\right)$7:**until** r≠0 **and** s≠08:**return** (r,s)

**Algorithm 13** 
ECDSA.Verify(pk,msg,(r,s))→b

1:

y←pk

2:**if** s=0 **or** r=0 **then**3:    **return** reject4:
**end if**
5:

$a\leftarrow \mathrm{Hash}\left(msg\right)\cdot s^{-1}\;\left(\mathrm{mod}\;q\right)$

6:

$b\leftarrow r\cdot s^{-1}\;\left(\mathrm{mod}\;q\right)$

7:

$y^{\prime }\leftarrow g^{a}\cdot y^{b}\;\left(\mathrm{mod}\;q\right)$

8:**if** $y^{\prime }$ is the point at infinity in *G* **then**9:    **return** reject10:
**end if**
11:**if** the *x*-coordinate of $y^{\prime }$ on *G* modulo *q* equals *r* **then**12:    **return** accept13:
**else**
14:    **return** reject15:
**end if**



### 2.6. Combiners and Hybrid Constructions

KEM combiners provide a method for constructing a new KEM by integrating two or more existing KEMs. The primary security goal of KEM combiners is to ensure that the resulting combined KEM remains secure even if one of the original KEMs is compromised. This robustness is particularly valuable in developing hybrid schemes that combine classical and post-quantum algorithms, thereby enhancing resilience against future vulnerabilities.

Several approaches to KEM combiners have been explored in the literature. Below, we mention two that satisfy the discussed security requirements for KEMs:

- XOR-then-MAC Combiner: In this approach, XOR, the outputs of the combined KEMs are utilized to generate an initial shared secret. The shared secret is then authenticated using a MAC to protect against mix-and-match attacks, where an adversary could combine parts of different ciphertexts to forge valid combinations, thus compromising more naive combiners;
- Dual-PRF Combiner: In this method, both KEMs are executed to produce a pair of secrets $\left(k_{1},k_{2}\right)$ and a pair of ciphertexts $\left(c_{1},c_{2}\right)$. The secrets are then combined using a pseudo-random function (PRF) to generate a key *k*, which is used to instantiate another PRF. The final secret is produced by running $PRF(k,\left(c_{1},c_{2}\right))$.

Both combiners are further analyzed, and their security is proven in [20]. A comprehensive analysis of KEM combiners and their security properties is provided by Giacon et al. [21], which formalizes the security definitions and constructions for these combiners. Their study emphasizes the importance of properly defining and constructing combiners to ensure the desired security guarantees.

In our proposal, we specifically evaluate the performance and security of the XOR-then-MAC combiner.

### 2.7. The KEMTLS Protocol

KEMTLS is a proposed protocol designed to establish secure connections using post-quantum algorithms, with the potential to replace the current TLS 1.3 standard. At a high level, the protocol operates as follows [6]:

1. C→S: “ClientHello”
  The Client sends a “ClientHello” message containing a newly generated ephemeral public key $pk_{e}$, created by running $KEM.KeyGen(1^{\lambda })$.
2. S→C: “ServerHello”
  The server responds with a “ServerHello” message that includes:
  - An encapsulated secret ($c_{e}$) generated using the KEM encapsulation function with the client’s public key ($pk_{e}$);
  - A certificate chain that includes the server’s public key ($pk_{S}$).
3. C→S: “ClientKeyExchange”
  The client replies with a “ClientKeyExchange” message containing $c_{S}$, which encapsulates a secret $k_{S}$ computed by running $KEM.Encaps(pk_{S})$.
  The final shared secret is derived by combining $k_{e}$ and $k_{S}$. With both secrets known, the client can begin sending encrypted application data alongside the “ClientKeyExchange” message, even before the handshake is fully completed.
4. S→C: “Finished”
  The server sends a “Finished” message to confirm the successful exchange of secrets. At this point, both parties establish the shared secret. Optionally, the server can send encrypted application data along with the “Finished” message.

Although KEMTLS requires two round trips to complete the full handshake—seemingly less efficient than the single round trip of TLS 1.3—this limitation can be mitigated. The client has the option to send application data concurrently with the “ClientKeyExchange” message, reducing overall latency before the handshake is fully completed.

Additionally, if the client already possesses the server’s public key, it can include the $c_{S}$ encapsulation in the initial “ClientHello” message. This allows the server to respond with both the “ServerHello” and the “Finished” message within the second round trip, achieving secret sharing in a single round trip.

While KEMTLS supports additional features such as client authentication, this paper focuses on the core mechanics of the protocol described above. The principles introduced can be extended to incorporate these additional functionalities as needed.

## 3. Our Hybrid KEMTLS Proposal

### 3.1. The Proposed Hybrid KEM Scheme (HKEM)

By combining public key KEMs with IDKEMs, we can leverage the strengths of both cryptographic primitives. Our hybrid scheme integrates a PKI-based KEM with an identity-based IDKEM using KEM combiners, creating a robust and flexible hybrid KEM. The hybrid scheme consists of the following algorithms:

- $HKEM.MasterKeyGen\left(1^{\lambda }\right)\to \left(mpk,msk\right)$: This algorithm generates the master public key mpk and master secret key msk, similar to IDKEM.KeyGen;
- $HKEM.KeyGen\left(1^{\lambda }\right)\to \left(pk,sk\right)$: Each user runs this algorithm locally to generate a public–private key pair (pk,sk), identical to KEM.KeyGen;
- $HKEM.Extract\left(msk,id\right)\to sk_{id}$: Given the master secret key msk and an identity id, this algorithm generates a secret key $sk_{id}$ corresponding to the identity, similar to IDKEM.Extract;
- HKEM.Encaps(mpk,pk,id)→(k,c): This probabilistic polynomial-time algorithm, given the master public key mpk, a public key pk, and an identity string id, produces a secret *k* and a ciphertext *c* that encapsulates the secret securely;
- $HKEM.Decaps(sk_{id},sk,c)\to k$: This deterministic polynomial-time algorithm, given the secret key associated with identity $sk_{id}$, another secret key sk, and a ciphertext *c*, recovers the secret *k* encapsulated in the ciphertext.

While the first three algorithms mirror those of the standard KEM and IDKEM schemes, the encapsulation and decapsulation algorithms must be specifically defined for the hybrid KEM. Following the XOR-then-MAC construction from [21], we define them in Algorithms 14 and 15.
**Algorithm 14** 
HKEM.Encaps(mpk,pk,id)
1:$\left(c_{1},k_{1a}\|k_{1b}\right)\leftarrow \mathrm{KEM}.\mathrm{Encaps}\left(pk\right)$2:$\left(c_{2},k_{2a}\|k_{2b}\right)\leftarrow \mathrm{IDKEM}.\mathrm{Encaps}\left(mpk,id\right)$3:$k_{kem}\leftarrow k_{1a}⊕k_{2a}$4:$k_{mac}\leftarrow k_{1b}\|k_{2b}$5:$c\leftarrow (c_{1},c_{2})$6:$t\leftarrow {\mathrm{MAC}}_{k_{mac}}\left(c\right)$7:**return** $\left(\left(c,t\right),k_{kem}\right)$

**Algorithm 15** 
$HKEM.Decaps(sk_{id},sk,\left(c,t\right))$

1:

$(c_{1},c_{2})\leftarrow c$

2:

$k_{1a}\|k_{1b}\leftarrow \mathrm{KEM}.\mathrm{Decaps}\left(sk,c_{1}\right)$

3:

$k_{2a}\|k_{2b}\leftarrow \mathrm{IDKEM}.\mathrm{Decaps}\left(sk_{id},c_{2}\right)$

4:

$k_{kem}\leftarrow k_{1a}⊕k_{2a}$

5:

$k_{mac}\leftarrow k_{1b}\|k_{2b}$

6:**if** $t\neq {\mathrm{MAC}}_{k_{mac}}\left(c\right)$ **then**7:    **return** ⊥8:
**else**
9:    **return** $k_{kem}$10:
**end if**



If we use a post-quantum identity-based KEM construction like DLP-IBE [17] and combine it with a classical Diffie–Hellman KEM, we can achieve a post-quantum construction without needing to modify existing keys or certificate chains, provided the key in the leaf certificate is compatible with Diffie–Hellman KEM (i.e., it must be an elliptic curve key rather than an RSA key). KEMTLS can then be used to perform the handshake. Our construction, based on HKEM, behaves like a typical hybrid KEMTLS construction, with the following differences:

- No post-quantum key required in the leaf certificate: We do not need to add a post-quantum key to the leaf certificate. In fact, we can continue using the same certificates in use today, provided they are compatible with DHKEM and we assume that classical signatures remain secure. Compared to a typical hybrid KEMTLS, the data sent during the “ServerHello” message will be smaller due to the reduced size of the certificate chain. Based on the public key sizes of the finalists in NIST’s Round 3 post-quantum standardization process, this would save approximately 672 to 800 bytes (NIST Security Level 1) or 930 to 1134 bytes (NIST Security Level 3).
- Ciphertext size in the “ClientKeyExchange” message: According to theoretical predictions from [22], the “ClientKeyExchange” message needs to send $N(2\left\lceil \log_{2}q\right\rceil -l)+mlen+hlen$ bits as ciphertext produced by the post-quantum part of HKEM. In this case, *N* is the number of dimensions of the underlying lattice, which must be at least 1024 to ensure 128 bits of security. The variable *l* is the compression parameter, which increases the probability of decryption failure only negligibly. According to [17], *l* should satisfy $l\leq \lfloor \log_{2}q\rfloor -3$, where *q* is the modulus in the Ring-LWE setting. Suggested values for *q* range from 23 bits [22] to 28 bits [23]. The variable mlen is the size of the internal message encrypted by DLP-IBE, and hlen is the digest size for the hash used in the Fujisaki–Okamoto transform to achieve IND-CCA security. Both mlen and hlen are typically 1280 (for 128-bit security) or 1408 (for 192-bit security).
  Thus, the ciphertext in our proposal is expected to be between 3488 and 4144 bytes, depending on the parameters suggested in the literature. By comparison, the ciphertext sizes for the NIST finalists range from 699 to 768 bytes (NIST Security Level 1) or 930 to 1088 bytes (NIST Security Level 3). This indicates that, unfortunately, using known realistic parameters, our proposal would produce larger ciphertexts compared to other KEMs being standardized by NIST. The increase in the size of the “ClientKeyExchange” message would outweigh the savings in the “ServerHello” message.
- Providing the PKG information: If the client does not already know which Private Key Generator (PKG) the server uses or its master public key, this information must be provided by the server. The server could send this information either within the leaf certificate or, if the existing certificate chains cannot be modified, as an additional signed message after the certificate chain. The size of the mpk is $N(\log_{2}q)$ bits, which would add between 2944 and 3584 bytes to the handshake based on current proposals for *N* and *q*. If the mpk is sent outside the leaf certificate, an additional ECDSA signature would be required for verification using the key in the leaf certificate.

### 3.2. Security Modelling for the Hybrid Construction

Typically, combiners mix two constructions of the same cryptographic primitive to create a new construction of the same type. Our approach differs: we combine two related but distinct primitives—public-key KEM and identity-based KEM—to create a new primitive that is neither a pure public-key KEM nor an identity-based KEM. Consequently, we require a new security definition for this hybrid primitive.

There are notable differences between the classical security model for PKI-based KEMs (Section 2.2) and identity-based KEMs (Section 2.3). Classical KEM security ensures that no adversary can break the security of a new key. In contrast, identity-based KEM security ensures that no adversary can break the security of any identity chosen by the adversary. The classical model operates in a single-user setting, while the identity-based model involves a multi-user setting. Strong security for public-key cryptography implies that schemes secure in the single-user setting are also secure in the multi-user setting [24]. If an adversary wants information about different users, they can simulate those users by generating their keys. However, this simulation is not possible in identity-based encryption. Therefore, we must allow the adversary to query oracles to accurately model its real-world capabilities. The same holds for our hybrid scheme, where identity-based KEM security serves as the basis for the hybrid construction.

Our hybrid model introduces several differences compared to traditional identity-based KEMs, which must be reflected in the security model. Each user in our model has an identity as well as a pair of local public and secret keys. Therefore, when modeling the attack game, we must maintain a dictionary to store each pair of keys along with their associated identities. Whenever an adversary queries information related to a specific identity, the associated keys must be generated and stored if they do not already exist.

Since public keys are not confidential, the adversary should be allowed to query the public key for any identity. Furthermore, the model must permit queries to compromise selected identities, reflecting real-world scenarios where an adversary might corrupt or control other users. After a compromise query, the adversary obtains both the local secret key sk and the identity secret key $sk_{id}$ for the selected identity.

This model also adapts decapsulation queries, allowing the adversary to specify the identity for which they seek decapsulation information.

The HKEM Attack Game, defined using Algorithms 16–19, illustrates the complete definition of the attack game for our hybrid KEM.
**Algorithm 16** HKEM Attack Game *b* (where *b* is either 0 or 1)1:Dict←EmptyDictionary()2:$\left(msk,mpk\right)\leftarrow \mathrm{HKEM}.\mathrm{MasterKeyGen}\left(1^{\lambda }\right)$3:$\left(id^{*},st\right)\leftarrow A_{1}^{\mathrm{Decaps}(msk,\cdot ),\mathrm{Compromise}(msk,\cdot ),\mathrm{PublicKey}(\cdot )}\left(mpk\right)$4:$pk^{*}\leftarrow \mathrm{PublicKey}\left(id^{*}\right)$5:$\left(c^{*},k_{0}\right)\leftarrow \mathrm{HKEM}.\mathrm{Encaps}\left(mpk,pk^{*},id^{*}\right)$6:$k_{1}\leftarrow R$           ▹ Randomly chosen key from key space *K*7:$b\leftarrow A_{2}^{\mathrm{Decaps}(msk,\cdot ),\mathrm{Compromise}(msk,\cdot ),\mathrm{PublicKey}(\cdot )}\left(st,c^{*},k_{b}\right)$8:**return** *b*

**Algorithm 17** Decapsulation Query for HKEM Attack Game
1:Decaps(msk,id,c):2:**if** Dict[id]≠∅ **then**3:    (pk,sk)←Dict[id]4:
**else**
5:    $\left(pk,sk\right)\leftarrow \mathrm{HKEM}.\mathrm{LocalKeyGen}\left(1^{\lambda }\right)$6:    Dict[id]←(pk,sk)7:
**end if**
8:

$sk_{id}\leftarrow \mathrm{HKEM}.\mathrm{Extract}\left(msk,id\right)$

9:**return** $\mathrm{HKEM}.\mathrm{Decaps}(sk_{id},sk,c)$


The adversary’s objective is to distinguish between the two scenarios. The adversary’s advantage is denoted as $Adv_{HKEM_{XtM}}^{IND-CCA}\left(A\right)=\left|\mathrm{Pr}\left[W_{0}\right]-\mathrm{Pr}\left[W_{1}\right]\right|$, where $W_{0}$ is the event in which it returns one in HKEM Attack Game 0, and $W_{1}$ is the event in which they return one in HKEM Attack Game 1. The adversary cannot use $id^{*}$ in a Compromise Query or $\left(id^{*},c^{*}\right)$ in a Decapsulation Query.

We can now present and prove a theorem, demonstrating that our hybrid construction utilizing the XOR-then-MAC combiner is secure under the security model established above.
**Algorithm 18** Compromise Query for HKEM Attack Game1:Compromise(msk,id):2:**if** Dict[id]≠∅ **then**3:    (pk,sk)←Dict[id]4:**else**5:    $\left(pk,sk\right)\leftarrow \mathrm{HKEM}.\mathrm{LocalKeyGen}\left(1^{\lambda }\right)$6:    Dict[id]←(pk,sk)7:**end if**8:$sk_{id}\leftarrow \mathrm{HKEM}.\mathrm{Extract}\left(msk,id\right)$9:**return** $\left(sk,sk_{id}\right)$

**Algorithm 19** Public Key Query for HKEM Attack Game
1:PublicKey(msk, id):2:**if** Dict[id]≠∅ **then**3:    (pk,sk)←Dict[id]4:
**else**
5:    $\left(pk,sk\right)\leftarrow \mathrm{HKEM}.\mathrm{LocalKeyGen}\left(1^{\lambda }\right)$6:    Dict[id]←(pk,sk)7:
**end if**
8:**return** pk

**Theorem** **1.** *We let $HKEM_{XtM}$ be a hybrid key encapsulation mechanism constructed using the XOR-then-MAC combiner, with a message authentication code MAC, a public-key key encapsulation mechanism KEM, and an identity-based key encapsulation mechanism IDKEM. If, for all adversaries $A_{1}$, $A_{2}$, and $A_{3}$, we determine that $Adv{KEM}^{IND-CCA}\left(A_{1}\right)$, $Adv{IDKEM}^{IND-CCA}\left(A_{2}\right)$, and $Adv{MAC}^{OT-sEUF}\left(A_{3}\right)$ are negligible, then for all adversaries $A_{4}$, $Adv_{HKEMXtM}^{IND-CCA}\left(A_{4}\right)$ is also negligible.*

The proof is provided in Appendix A. We emphasize that our security proof follows the same structure and steps as those in [21], with adaptations made to accommodate our slightly different security definition.

### 3.3. The Full Proposal and General Security Discussion

By integrating our hybrid KEM into the KEMTLS protocol alongside a post-quantum KEM with ephemeral keys generated by the client, we can create a hybrid KEMTLS that does not require including a post-quantum key in the certificate. However, we still need to indicate which Private Key Generators (PKGs) the server supports, either by extending the certificate or modifying the protocol. The server may also provide a list of supported PKGs, allowing the client to choose one it trusts for encapsulation with the hybrid KEM.

If we select a Diffie–Hellman KEM as the classical component of our hybrid construction, the public key in the certificate will be an elliptic curve point, similar to TLS 1.3. Therefore, in the case of a new certificate, the server can generate a Certificate Signing Request (CSR) using that elliptic curve point as the public key and submit it to any existing certificate authority.

CSRs require proof of knowledge, wherein applicants sign the message using the secret key corresponding to the public key being certified, typically with ECDSA. This ensures that the applicant possesses the matching secret key. However, this process poses a risk to our proposal: unlike TLS 1.3, the keys in our construction are part of a KEM, not a signature scheme. Using the same keys for signing messages and later for secret exchange in a KEM may compromise the security of both the signature scheme and the KEM.

While not all combinations of signature schemes and KEMs can securely share keys, we demonstrate that using the same pair of keys for both an ECDSA signature and a DHKEM does not compromise the security of either cryptographic primitive. This analysis is challenging due to the lack of strong security guarantees for ECDSA signatures. Recent findings in [25] suggest that proving the security of ECDSA without relying on idealized models, such as the generic group or random oracle models, is infeasible. Nevertheless, various models in the literature reduce the security of ECDSA to solving the discrete logarithm problem or similar problems.

We model the ECDSA signature as in [26], where only Line 5 of the signing algorithm, as described in Section 2.5, is replaced by an idealized function. Our analysis shows that the security proofs for both ECDSA signatures and Diffie–Hellman KEM do not interfere with each other, even when these primitives share the same key.

We arrive at this conclusion by modeling security using two new SIG-KEM Attack Games: Game 0 and Game 1, described as Algorithms 20 and 21. These combine the Signature Attack Game with the KEM Attack Games, similar to how the two games define IND-CCA security for KEMs. The difference here is that the adversary can now query for signatures, assuming the signature scheme and KEM share the same keys. At the end of the game, the adversary outputs a bit *b* and a pair (msg∗,sig∗). We let $W_{0}$ represent the event where the adversary A outputs b=1 in SIG-KEM Game 0, and $W_{1}$ the event where it outputs b=1 in SIG-KEM Game 1. The advantage of a given adversary in the SIG-KEM game is the greater of $|W_{0}-W_{1}|$ or the probability of producing a valid forgery for a new message using the ECDSA signature in either game.
**Algorithm 20** SIG-KEM Attack Game 01:$\left(sk,pk\right)\leftarrow \mathrm{KeyGen}\left(1^{\lambda }\right)$   ▹ Key generation shared between KEM and signature.2:Q←∅      ▹*Q*: set of messages sent to signing queries, initialized empty3:$\left(c^{*},k_{0}\right)\leftarrow \mathrm{KEM}.\mathrm{Encaps}\left(pk\right)$4:$\left(b,msg*,sig*\right)\leftarrow A^{\mathrm{KEM}.\mathrm{Decaps}(sk,\cdot ),\mathrm{SigningQuery}(sk,\cdot )}\left(pk,c^{*},k_{0}\right)$5:**if** $SIG.Verify(pk,msg^{*},sig^{*})=\mathrm{accept}$ **and** $msg^{*}\notin Q$ **then**6:    **return** (b,1)7:**else**8:    **return** (b,0)9:**end if**

**Algorithm 21** SIG-KEM Attack Game 1
1:

$\left(sk,pk\right)\leftarrow \mathrm{KeyGen}\left(1^{\lambda }\right)$

2:

Q←∅

3:

$\left(c^{*},-\right)\leftarrow \mathrm{KEM}.\mathrm{Encaps}\left(pk\right)$

4:$k_{1}\;\overset{\;\$}{\leftarrow }\;K$          ▹ Randomly chosen key from key space *K*5:

$\left(b,msg*,sig*\right)\leftarrow A^{\mathrm{KEM}.\mathrm{Decaps}(sk,\cdot ),\mathrm{SigningQuery}(sk,\cdot )}\left(pk,c^{*},k_{1}\right)$

6:**if** $SIG.Verify(pk,msg^{*},sig^{*})=\mathrm{accept}$ **and** $msg^{*}\notin Q$ **then**7:    **return** (b,1)8:
**else**
9:    **return** (b,0)10:
**end if**

**Theorem** **2.** *Assuming the computational Diffie–Hellman problem is hard and that we can efficiently verify solutions to the problem, no adversary can achieve a non-negligible advantage in the SIG-KEM Attack Games when we instantiate the signature with ECDSA and the KEM with DHKEM.*

The proof is provided in Appendix B. Note that, since our proposal involves the possible sharing of keys between these distinct primitives only in the classical part of the hybrid construction, the Diffie–Hellman assumption is acceptable despite not being post-quantum.

Furthermore, as shown in the proof, information revealed by decapsulation queries does not interfere with the results or probabilities when computing responses for signing queries. Similarly, the signatures produced do not interfere with decapsulation queries, thanks to the use of different random oracles for each query type. The upper bound on the probability of breaking the KEM or the ECDSA signatures when they share the same pair of keys is simply the sum of the probabilities of an adversary breaking either the ECDSA or the KEM.

Additionally, similar proofs can be derived using alternative models for ECDSA security. Some works, such as [27,28], prove the security of ECDSA signatures in the generic group model. A security proof for key sharing between ECDSA signatures and DHKEM could also be made in that model, but it requires defining the gap Diffie–Hellman assumption in the generic model and extending the oracle to allow adversaries to query whether a triple of group elements forms a Diffie–Hellman triple.

Another model of ECDSA signature security is presented in [29], where the security proof assumes that each message is not signed twice (an assumption compatible with TLS). However, this security reduction is based on a weaker and non-standard assumption compared to the computational Diffie–Hellman problem. Using that model, a similar security proof to the one in the appendix could be derived, as that work simulates ECDSA signatures similarly. Adapting that proof, however, requires both the computational Diffie–Hellman assumption and the weaker intractable semi-logarithm assumption discussed in the article.

The security reduction from [29] applies not only to ECDSA and DSA signatures but also to Chinese SM2 [30] and Russian GOST [31] signatures, suggesting that our proposal could provide equivalent security for these signature schemes as well.

### 3.4. Migrating to Post-Quantum TLS

To provide a practical illustration of how our proposal can be used during migration, we compare it with existing proposals like KEMTLS and PQ-TLS. We also discuss some potential use cases in future scenarios.

Migrating existing systems to post-quantum protocols, such as KEMTLS or PQ-TLS, requires updates to the TLS protocol on clients, servers, and CAs. These entities must support the new algorithms. Additionally, certificate authorities must adapt their CSRs, since the current proof of knowledge—where a server proves possession of the secret key associated with the public key—assumes that the certified key belongs to a signature scheme, not a KEM. For post-quantum KEMs, proof of knowledge needs a new interactive protocol or a non-interactive zero-knowledge proof (as proposed for lattice-based KEMs in [32]).

In contrast, our proposal requires only that the client and server update their protocol; certificate authorities do not need to change, and the same protocols can be used. Instead, the protocol includes the presence of a PKG, which acts as the central authority that distributes the secret keys associated with the post-quantum IBE used in our KEM. This distribution must be authenticated.

Additionally, while existing KEMTLS and PQ-TLS proposals require servers to generate new keys and re-certify post-quantum keys, our approach allows the continued use of existing classical keys and certificates.

**Figure 3:**
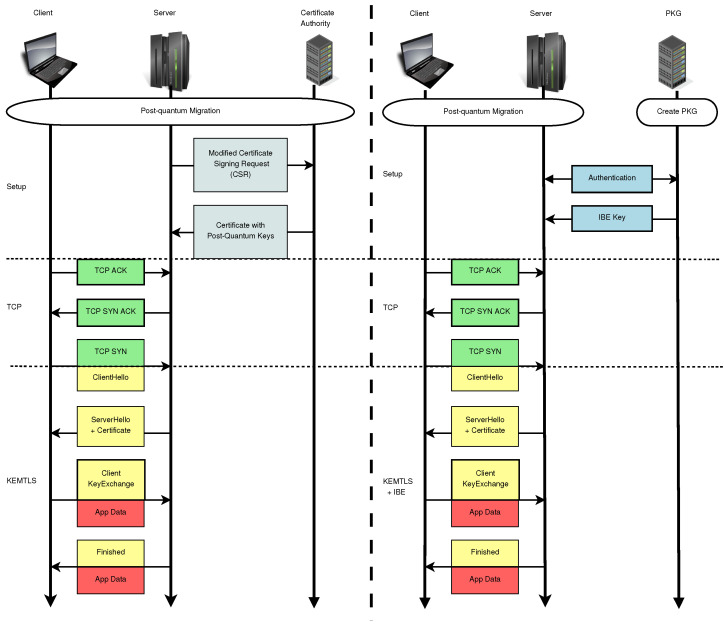
Side-by-side comparison between KEMTLS (**left**) and our proposal (**right**). Our approach does not require changes in the certificate authority or new post-quantum certificates. If new keys are needed, we can use the same CSRs as in TLS 1.3, whereas typical KEMTLS needs to adapt.

**Figure 4:**
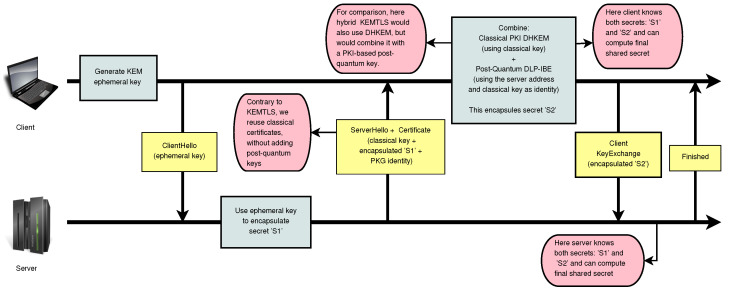
A more detailed view of our proposal mixing KEMTLS and IBE. The yellow boxes represent the messages sent between the client and server over the network. The gray boxes indicate computations performed locally by both the client and server. The red boxes provide additional comments on key aspects of the protocol and emphasize the differences between this proposal and a typical hybrid construction using KEMTLS.

Figure 3 illustrates the migration process, comparing a KEMTLS protocol migration (left) with our proposal (right). Figure 4 offers more details about how our modified KEMTLS works, stressing the differences with a more typical hybrid version of KEMTLS.

The specific details of the migration process depend on the scenario. Below are some example use cases.

- IoT devices: A manufacturer has devices in the market that use TLS to communicate with a server. Through software and firmware updates, the manufacturer can update all involved components, centralizing the PKG responsibility. Devices can receive the PKG’s public key via updates, while the server receives its new secret key for the IBE. Since existing certificates need not be changed, the migration can proceed even if certificates are stored in ROM and cannot be updated;
- Centralized service: An operating system manufacturer, or a group of manufacturers, offers our protocol as a service that provides automatic post-quantum protection to servers. Server administrators need only subscribe to the service; no new keys need to be generated. Multiple PKGs may exist, but limited in number. The PKG’s public key can be identified by an associated number, which is signed with the server’s classical key and included in the “ServerHello” message;
- Decentralized PKGs: If the migration is not centrally managed, multiple independent PKGs may emerge. Clients and servers can migrate via operating system updates or by installing specific applications. Two trust models are possible: (1) the client can trust the server’s identification of the PKG via a signed message, allowing the server to certify the PKG for the client; or (2) the PKG must be certified by an authority trusted by both the client and the server. In this latter case, the certificate needs to be extended to include both the server’s and the PKG’s identification, which negates the advantage of using existing certificates.

## 4. Experiments

To evaluate the performance and feasibility of our proposed hybrid KEMTLS, we modified the KEMTLS implementation from [33] to incorporate the hybrid construction detailed in Section 3. For the identity-based post-quantum KEM, we adopted the DLP-IBE scheme from [17] using the implementation provided in [16]. The classical DHKEM implementation was sourced from the Circl library [34]. Additionally, we constructed a hybrid KEM combining Kyber [35] with DHKEM for ephemeral key encapsulation. The combiner logic and the necessary KEMTLS adaptations were implemented in the Go programming language [36].

Our proof-of-concept implementation took the form of a modified version of the Go standard library. This modified library can be linked to any Go program that uses TLS, enabling it to use our proposed hybrid KEMTLS. However, the program must explicitly specify that it wants to use the modified TLS instead of TLS 1.3, and it must also configure the connection with the relevant PKG information. Along with this modification of the TLS protocol, we developed tests and benchmarks integrated into the library’s test suite, which were used to perform the performance measurements described below.

Our proposal offers three distinct deployment scenarios based on the level of information exchange between the server and client:

- KEMTLS-IBE-PDK: In environments where the client already possesses the necessary server keys, we use KEMTLS with pre-distributed keys (PDKs) as described in [37]. This scenario applies when the client and server have previously established a connection, or in IoT cases where a device always connects to the same server. This configuration enables a single-round handshake without requiring certificate chains or the IBE master public key (mpk).
- KEMTLS-IBE: When the IBE mpk is disseminated via out-of-band channels, we use the standard KEMTLS protocol.
- KEMTLS-IBE-MPK: For cases where the IBE mpk must be communicated during the handshake, we introduce an additional signed message following the certificate chain. This message is signed using the P256 key embedded in the leaf certificate via ECDSA.

Our initial experiment aimed to assess the performance impact of transitioning from TLS 1.3 to our proposed solution without altering the underlying TLS infrastructure. To establish a baseline, we constructed a certificate chain comprising a self-signed RSA-4096 root certificate (using SHA-384), an intermediate RSA-2048 certificate (hashed with SHA-256), and a leaf certificate with a 256-bit elliptic curve key (also hashed with SHA-256). This certificate chain configuration mirrors common internet practices, including Google’s web pages, and was used for our experiments. For the KEMTLS-IBE-MPK configuration, which requires sending the PKG mpk during transmission, we sent it as an additional signed message along with the “ServerHello” message, as described in the last use case in Section 3.4.

To compare handshake sizes, we measured the total number of bytes exchanged between the client and server, encompassing all messages. Table 1 presents the results. Note that the total handshake size slightly exceeds the sum of individual message sizes due to the inclusion of the final “Finished” message. The same certificate chain, sized at 2050 bytes, was used for all protocols except KEMTLS-IBE-PDK, as protocols with pre-distributed keys do not require certificates.

**Figure 5:**
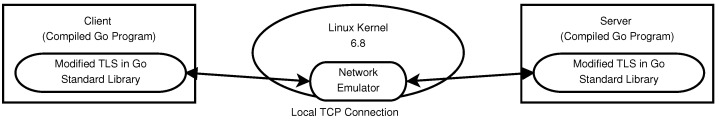
Setup for our local tests in a simulated environment. Since our tests were integrated into the Go standard library, both the client and server were Go executables, each running a different test function. By modifying a configuration structure in the source code, we were able to use various protocols over the local TCP connection, including TLS 1.3, KEMTLS, and different variations of our proposed KEMTLS-IBE. To simulate a high-bandwidth channel with different latencies, we invoked the “tc qdisc” command with different parameters before running our tests, configuring the network emulator in the Linux kernel.

To evaluate performance under simulated network conditions, we employed Linux Network Emulator (NetEM) [38] on a local machine running Linux 6.8 (Ubuntu 22.04.4, produced by Canonical Ltd., downloaded from official web page) with an Intel i5 3.10 GHz 8-core processor, shipped in a Lenovo Ideapad Notebook 82G0009BR, manufactured by Lenovo Group Limited in Indaiatuba, Brazil. We measured handshake time in milliseconds by timing the complete TLS handshake process. Network simulations imposed a 1000 Mbit/s bandwidth cap and varied packet delays between 0 ms and 150 ms. Table 2 summarizes the results, presenting mean and standard deviation values for handshake times across different protocols and latency settings. Additionally, the table includes measurements from a real-world network scenario for comparison. The setup for the tests in simulated environments is shown in Figure 5.

Directly comparing handshake times between KEMTLS and TLS 1.3 is challenging, as KEMTLS involves an additional round-trip. However, both protocols allow application data to be sent after the first round-trip. To address this, we use the “Time-to-send-app-data” (or “SendApp Time”) metric introduced in [8], which measures the delay before the client can send application data. While TLS 1.3 and KEMTLS-IBE-PDK show similar times for both the full handshake and the SendApp Time, KEMTLS-IBE and KEMTLS-IBE-MPK demonstrate significantly shorter SendApp Times, as they allow application data to be sent before the handshake is fully completed.

**Figure 6:**
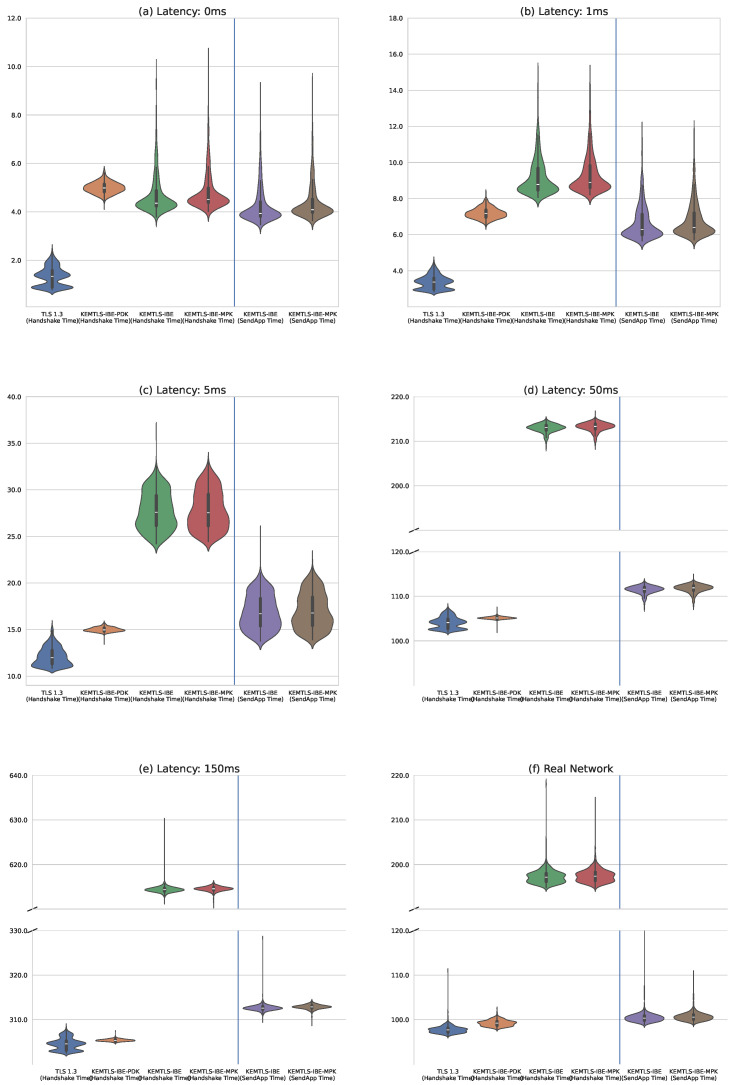
Comparison of our proposal with TLS 1.3. All timings are in milliseconds. (**a**) Running the protocols over a local connection without introducing any artificial latency using NetEM. (**b**) Introducing a latency of 1 ms with NetEM. (**c**) Latency of 5 ms introduced with NetEM. (**d**) Latency of 50 ms. (**e**) Latency of 150 ms. (**f**) Measuring timings over a real internet connection.

Figure 6 provides a visual representation of the handshake and SendApp time data using violin plots, illustrating the distribution of these values across different protocols and latency conditions. The first subplot shows the timings when the protocol is run locally, without introducing delays with NetEM. The following four subplots progressively introduce higher delays to simulate greater latencies.

**Figure 7:**
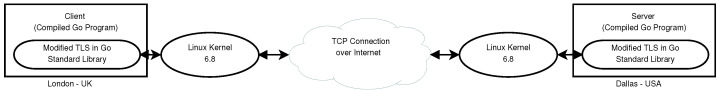
Setup for remote experiments over a real network. We used the same client and server configuration as in the simulated environment and ran the same tests. However, the client and server were deployed on different rented machines across different continents. Instead of configuring the Linux network emulator, we connected them over a real internet connection using different TLS protocols.

To conduct a more realistic evaluation, we deployed a client in London, UK and a server in Dallas, USA on virtual machines hosted by different providers, as shown in Figure 7. While the measured latency between these machines ranged from 40 ms to 50 ms, we acknowledge that internet connection dynamics may vary. We executed 1000 handshake iterations for each protocol, recording the full handshake time in milliseconds. The results from these real-world tests are included in the final row of Table 2 and the last subplot of Figure 6.

The data show that using our protocol, clients need to wait between 2.6% and 7.1% longer before being able to send application data when connecting to a remote server with latencies typical of an Internet connection (≥50 ms). The difference is larger in low-latency environments, likely because the slower algorithms used by IBE have a greater impact in such scenarios. Additionally, the data indicate that in high-bandwidth scenarios, sending or not sending an additional master public key (mpk) has minimal impact on overall performance.

To further evaluate how our proposal compares with other hybrid KEMTLS constructions, we conducted a second set of tests simulating a future TLS infrastructure that is fully resistant to quantum attacks. In this scenario, secret sharing and authentication are exclusively handled by hybrid KEMTLS schemes, and certificate chains are updated to use hybrid signature algorithms. The leaf certificate may optionally include an IBE master public key (mpk) for KEMTLS-IBE-MPK, as described in the last use case in Section 3.4.

We selected several NIST Level 3 post-quantum KEMs for testing. This security level aligns with the DLP-IBE scheme, which requires a power-of-two value for the lattice dimension parameter N [17]. A lattice dimension of 512 offers approximately 80-bit security (below NIST Level 1), while increasing N to 1024 yields around 192 bits of security, suitable for NIST Level 3. Although some studies, such as [22], explore modifications for 128-bit security, these changes have minimal impact on ciphertext size and do not affect the master public key length. Therefore, NIST Level 3 is ideal for leveraging this IBE construction.

To comprehensively assess our proposal’s performance in a fully post-quantum TLS environment, we conducted a second set of experiments. We combined various post-quantum KEMs with classical DHKEM to create different hybrid KEMTLS configurations. Simulating a future post-quantum ecosystem, we incorporated post-quantum certificate chains using a hybrid signature scheme combining ECDSA and the Dilithium-3 post-quantum signature [39]. These certificate chains consist of a root (omitted during transmission), an intermediate, and a leaf certificate. Table 3 summarizes the cumulative handshake message sizes for these configurations. The experimental methodology follows the approach in [8] for comparative analysis.

Mirroring our previous evaluation, we conducted experiments on a local machine simulating networks with varying latencies and packet loss. Each scenario included 1000 handshake measurements, with mean and standard deviation calculated. Additionally, handshake timings were captured for a client and server connected over the Internet, geographically separated.

**Figure 8:**
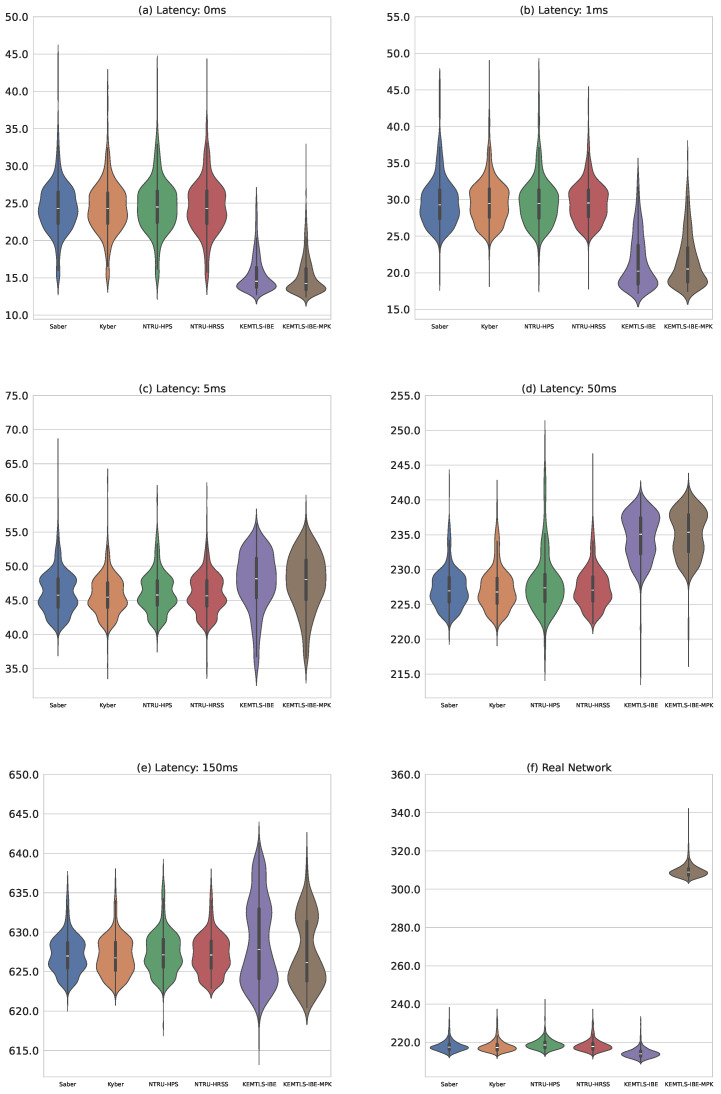
Comparison of our proposal with other hybrid algorithms. All timings are in milliseconds. (**a**) Running the protocols over a local connection without introducing any artificial latency with NetEM. (**b**) Introducing a latency of 1 ms with NetEM. (**c**) Latency of 5 ms introduced with NetEM. (**d**) Latency of 50 ms introduced. (**e**) Latency of 150 ms. (**f**) Measuring the timings over a real Internet connection.

Figure 8 presents violin plots comparing our proposal’s handshake times with other hybrid KEMTLS algorithms under various latency conditions. Each subplot corresponds to a specific latency setting. The first subplot shows the algorithms running locally without any artificial delay as in the previous tests. The next four subplots represent increasing latencies simulated with NetEm, and the final subplot shows timings for client–server connections over the Internet, with geographically distant locations. Detailed data for all configurations are available in Table 4.

A surprising outcome was the shorter handshake times for KEMTLS-IBE compared to other hybrid KEMTLS constructions, despite its larger message sizes. However, these performance differences were minimal, smaller even than variations observed in local, latency-free environments. This suggests that while KEMTLS-IBE may incur some network overhead, its computational efficiency compensates, resulting in faster overall handshake times. Simulation data indicate that increased latency could potentially reverse this trend, favoring other hybrid algorithms.

Another notable result is that, while not observed in simulation, KEMTLS-IBE-MPK performed significantly worse in a real network. We speculate that adding the mpk to the certificate crossed a certain threshold, making the message too large to transfer at comparable speeds.

**Figure 9:**
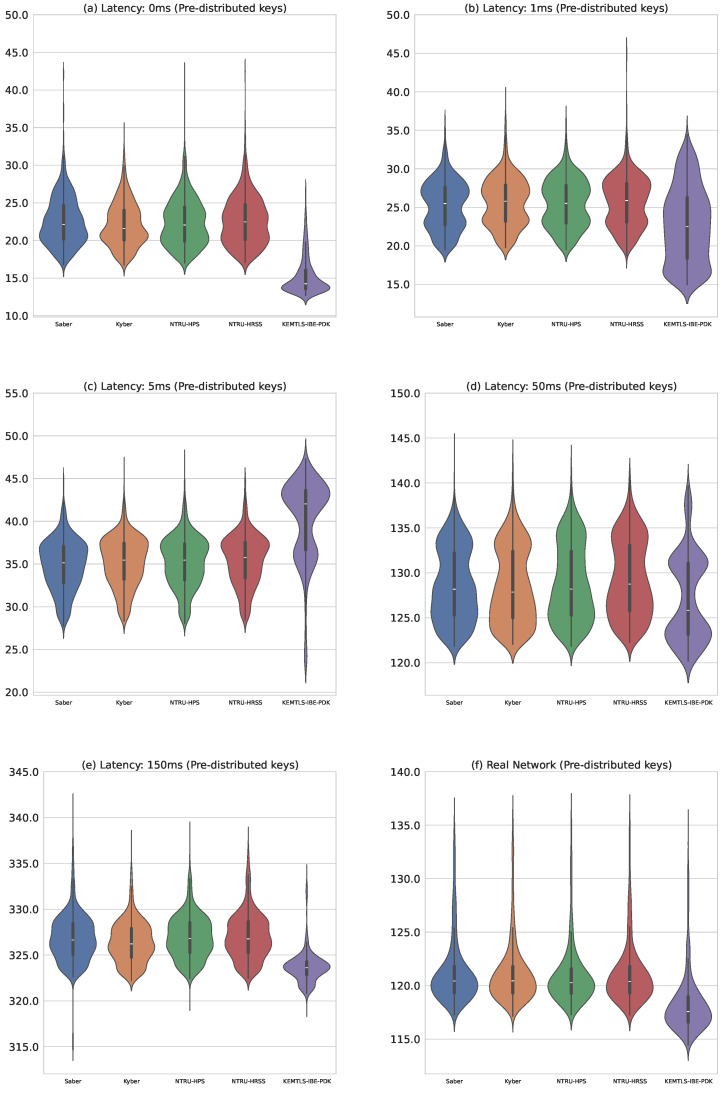
Comparison of our proposal with other hybrid algorithms using pre-distributed keys. All timings are in milliseconds. (**a**) Running the protocols over a local connection without introducing any artificial latency using NetEM. (**b**) Introducing a latency of 1 ms with NetEM. (**c**) Latency of 5 ms introduced with NetEM. (**d**) Latency of 50 ms introduced. (**e**) Latency of 150 ms. (**f**) Measuring timings over a real Internet connection.

We also evaluated the performance of KEMTLS-IBE-PDK, a variant that uses pre-distributed keys, against other hybrid KEMTLS constructions with pre-distributed keys. Table 5 compares handshake message sizes for these configurations, while Table 6 and Figure 9 compare the timings. As in the previous graphs, the first subplot represents algorithms running without artificial delays on the same machine, the next four subplots introduce increasingly higher delays using NetEm, and the final subplot shows timings over a real internet connection between distant geographic locations.

Despite the larger message sizes, our proposal demonstrated shorter handshake times under certain simulated and real-world network conditions. This unexpected result is likely due to the minor impact of network latency compared to the computational efficiency of our implementation. In the pre-distributed key scenario, algorithmic optimizations appear to play a more significant role in determining overall performance. Further research is required to evaluate all algorithms under equivalent optimization levels to establish a more definitive comparison.

## 5. Conclusions

This paper presents a hybrid Key Encapsulation Mechanism (KEM) for TLS 1.3, combining classical public key infrastructure (PKI) with identity-based encryption (IBE). Our approach aims to ease the transition to post-quantum cryptography by minimizing disruptions to existing TLS infrastructure and certificate management practices.

Summary of Contributions

- Hybrid KEMTLS Protocol: We introduce a novel hybrid KEMTLS protocol that integrates traditional public-key cryptography with post-quantum identity-based encryption. This approach enhances security by providing resilience against both classical and quantum adversaries;
- Practical Implementation: Our implementation demonstrates the feasibility of achieving post-quantum security without requiring immediate modifications to existing TLS infrastructure. By leveraging identity-based encryption, we streamline key management and reduce dependency on certificate authorities;
- Rigorous Security Analysis: We perform a comprehensive security analysis, demonstrating the hybrid KEMTLS protocol’s resistance to various attacks, including chosen-ciphertext attacks. The use of combiners ensures security even in the presence of vulnerabilities in individual components;
- Performance Evaluation: Through extensive experimentation, we show that our hybrid KEMTLS protocol delivers comparable performance to existing post-quantum solutions while minimizing disruptions to the current TLS ecosystem.

Implications for TLS Infrastructure

Integrating identity-based encryption into TLS 1.3 offers several practical benefits:

- Minimal Infrastructure Impact: Our hybrid approach requires minimal changes to existing TLS infrastructure. By deriving keys from server identities, we eliminate the need for post-quantum key embedding in certificates, ensuring a smoother transition to post-quantum security;
- Scalability and Compatibility: The protocol integrates seamlessly with existing PKI systems, enabling organizations to adopt post-quantum security incrementally without overhauling their cryptographic infrastructure;
- Enhanced Security Posture: By combining classical and post-quantum cryptography, we strengthen the overall security posture, ensuring resilience even if vulnerabilities are found in individual cryptographic components.

Future Work

Building on this research, several avenues for future work can be explored to further improve the hybrid KEMTLS protocol:

- IBE Scheme Optimization: Investigate techniques to optimize IBE schemes, focusing on reducing computational overhead and ciphertext sizes, and exploring novel constructions to improve protocol performance;
- Integration of Emerging Post-Quantum Algorithms: Expand the hybrid KEMTLS protocol to support emerging post-quantum algorithms, such as lattice-based and code-based schemes, to create more versatile and resilient solutions;
- Client Authentication: Extend the protocol to support client authentication using identity-based signatures or other post-quantum signature mechanisms, enabling comprehensive post-quantum security for both parties;
- Scalability and Deployment Analysis: Conduct in-depth studies to evaluate the protocol’s performance and scalability in large-scale environments, including cloud and distributed systems, identifying potential bottlenecks and optimization opportunities;
- Advanced Security Measures: Explore the integration of multi-factor authentication and hardware-based security components to further enhance the protocol’s security;
- Application Impact Assessment: Evaluate the impact of the protocol on existing TLS-dependent applications and services and develop best practices for smooth adoption;
- Interoperability with Classical TLS: Design mechanisms to ensure seamless interoperability between the hybrid KEMTLS protocol and traditional TLS systems, facilitating gradual adoption and providing fallback options.

The proposed hybrid KEMTLS protocol represents a significant step toward achieving post-quantum security within the TLS framework. By combining traditional PKI with identity-based encryption, we offer a practical and efficient solution for organizations transitioning to a post-quantum future. Our research addresses current security challenges while laying the groundwork for future advancements in secure communications.

This work demonstrates that post-quantum cryptographic solutions can be integrated into existing systems, facilitating widespread adoption. The hybrid KEMTLS protocol provides a robust and scalable approach to securing digital communications in an evolving threat landscape.

## Figures and Tables

**Table 1:** Handshake message sizes (in bytes).

Protocol	ClientHello	ServerHello	ClientKeyExchange	Total
TLS 1.3	385	2325	-	2746
KEMTLS-IBE-PDK	5291	974	-	6301
KEMTLS-IBE	1179	2979	4109	8303
KEMTLS-IBE-MPK	1179	6639	4109	11,963

**Table 2:** Handshake timings in miliseconds.

Delay	TLS 1.3		KEMTLS-IBE-PDK		KEMTLS-IBE				KEMTLS-IBE-MPK			
	**Handshake Time**		**Handshake Time**		**Handshake Time**		**SendApp Time**		**Handshake Time**		**SendApp Time**	
	**Mean**	**St.Dev.**	**Mean**	**St.Dev.**	**Mean**	**St.Dev.**	**Mean**	**St.Dev.**	**Mean**	**St.Dev.**	**Mean**	**St.Dev.**
0	1.32	0.39	4.99	0.23	4.70	0.80	4.25	0.74	4.84	0.81	4.40	0.75
1	3.36	0.37	7.19	0.29	9.21	1.05	6.68	0.96	9.35	1.08	6.83	0.99
5	12.10	0.98	15.00	0.24	27.77	2.00	16.87	1.83	27.85	2.07	17.00	1.89
50	104.08	1.29	105.16	0.28	212.92	0.97	111.47	0.90	213.20	1.01	111.74	0.94
150	304.53	1.32	305.38	0.26	614.37	0.75	312.63	0.73	614.53	0.57	312.85	0.55
Real	97.74	0.84	99.23	0.70	197.28	1.69	100.49	1.30	197.51	1.66	100.70	1.05

**Table 3:** Handshake size (in bytes) comparing our Hybrid KEMTLS construction with traditional Hybrid KEMTLS protocols. All scenarios utilize a hybrid combination of DHKEM and a post-quantum KEM.

Hybrid Algorithms	ClientHello	ServerHello	ClientKeyExchange	Total
DHKEM + Saber	1379	11,798	1225	14,438
DHKEM + Kyber	1571	11,994	1225	14,826
DHKEM + NTRU HPS	1317	11,581	1067	14,001
DHKEM + NTRU-HRSS	1525	11,996	1239	14,832
KEMTLS-IBE	1595	10,781	4109	16,596
KEMTLS-IBE-MPK	1595	14,384	4109	20,124

**Table 4:** Handshake time (in milliseconds) for various hybrid KEMTLS configurations.

Latency	DHKEM + Saber		DHKEM + Kyber		DHKEM + NTRU-HPS		DHKEM + NTRU-HRSS		KEMTLS-IBE		KEMTLS-IBE-MPK	
	**Mean**	**St.Dev.**	**Mean**	**St.Dev.**	**Mean**	**St.Dev.**	**Mean**	**St.Dev.**	**Mean**	**St.Dev.**	**Mean**	**St.Dev.**
0	24.43	3.60	24.39	3.47	24.60	3.65	24.55	3.65	15.47	2.48	15.18	2.45
1	29.76	3.47	29.68	3.06	29.73	3.47	29.68	2.81	21.46	3.68	21.56	3.50
5	46.17	3.14	45.72	2.82	46.14	2.96	45.94	2.89	47.70	4.38	47.67	4.25
50	227.24	2.76	227.24	3.06	228.06	4.39	227.39	2.88	234.61	3.53	235.06	3.39
150	627.14	2.38	626.96	2.43	627.41	2.65	627.22	2.48	628.82	5.32	627.38	4.34
Real	217.99	2.85	217.96	3.11	219.00	2.81	218.38	3.10	214.32	2.58	309.44	3.18

**Table 5:** Handshake size (in bytes) comparing our Hybrid KEMTLS construction with traditional Hybrid KEMTLS protocols using pre-distributed keys.

Hybrid Algorithms	ClientHello	ServerHello	Total
DHKEM + Saber (PDK)	2601	1331	3963
DHKEM + Kyber (PDK)	2793	1326	4185
DHKEM + NTRU HPS (PDK)	2381	1168	3585
DHKEM + NTRU-HRSS (PDK)	2797	1376	4209
KEMTLS-IBE-PDK	5707	1331	7033

**Table 6:** Handshake time in milliseconds when server keys were pre-distributed.

Latency	Saber (PDK)		Kyber (PDK)		NTRU-HPS (PDK)		NTRU-HRSS (PDK)		KEMTLS-IBE-PDK	
	**Mean**	**St.Dev.**	**Mean**	**St.Dev.**	**Mean**	**St.Dev.**	**Mean**	**St.Dev.**	**Mean**	**St.Dev.**
0	22.55	3.18	22.13	2.91	22.38	3.08	22.60	3.20	15.26	2.47
1	25.26	3.13	25.67	3.14	25.48	3.09	25.72	3.25	22.66	4.88
5	34.82	2.92	35.19	2.87	35.20	2.95	35.44	2.94	40.26	4.66
50	128.64	4.03	128.55	4.21	128.87	4.25	129.37	4.23	127.19	4.79
150	326.82	2.63	326.40	2.26	326.95	2.35	327.06	2.56	323.71	1.79
Real	121.29	3.15	121.14	2.96	120.93	2.86	121.10	2.94	118.27	2.84

## Data Availability

Data and source code for our experiments can be obtained in [40].

## Appendix A

**Proof** **of Theorem 1.** The proof consists of two steps. First, we assume that the classical Key Encapsulation Mechanism (KEM) is stronger than the identity-based KEM (IDKEM). In the second step, we assume the IDKEM is stronger. From these two cases, we conclude that in both scenarios, the security of the hybrid KEM is equivalent to the security of the strongest component. This means that even if one of the primitives is broken, the hybrid construction remains secure due to the strength of the other. In both steps, the proof follows the standard hybrid argument, commonly used in indistinguishability proofs. We demonstrate that an adversary cannot distinguish between two different scenarios by gradually transforming one scenario into another through a series of intermediate “hybrid” scenarios. We show that an adversary can detect these gradual changes only if they are capable of breaking one of the security assumptions (either of the KEM or IDKEM). Step 1: Assuming that KEM is safer than IDKEM. In this step, we assume that the classical KEM is more secure than IDKEM. To prove that the security of our hybrid KEM remains as strong as the KEM in this case, we introduce hybrid games that transition between the two scenarios. We gradually replace real keys with random ones, demonstrating that an adversary cannot distinguish between these scenarios unless they can break the underlying KEM. Hybrid Game 2 and 3: This game is described below and using Algorithm 17. It lies between hybrid KEM Game 0 and hybrid KEM Game 1. In Game 2, we replace the real key produced by KEM.Encaps with a randomly chosen uniform key while keeping the ciphertext $c_{1}$ intact. However, the key produced by IDKEM.Encaps remains unchanged. To ensure consistent behavior, the decapsulation query returns the same random value whenever it decapsulates $c_{1}$.

**Algorithm A1** Hybrid Attack Game 2 and 3
1: $id^{*}\leftarrow \emptyset$ 2: Dict←EmptyDictionary() 3: $\left(msk,mpk\right)\leftarrow \mathrm{HKEM}.\mathrm{MasterKeyGen}\left(1^{\lambda }\right)$ 4: $\left(id^{*},st\right)\leftarrow A_{1}^{\mathrm{Decaps}(msk,\cdot ),\mathrm{Compromise}(msk,\cdot ),\mathrm{PublicKey}(\cdot )}\left(mpk\right)$ 5: $pk^{*}\leftarrow \mathrm{PublicKey}\left(id^{*}\right)$ 6: $\left(c_{1},k_{1a}\|k_{1b}\right)\leftarrow \mathrm{KEM}.\mathrm{Encaps}\left(pk^{*}\right)$ 7: $\left(c_{2},k_{2a}\|k_{2b}\right)\leftarrow \mathrm{IDKEM}.\mathrm{Encaps}\left(mpk,id^{*}\right)$ 8: $k_{1a}\|k_{1b}\;\overset{\;\$}{\leftarrow }\;K$ ▹ Randomly chosen key from key space *K* 9: $k_{kem}\leftarrow k_{1a}⊕k_{2a}$ 10: $k_{mac}\leftarrow k_{1b}\|k_{2b}$ 11: $c\leftarrow (c_{1},c_{2})$ 12: $t\leftarrow {\mathrm{MAC}}_{k_{mac}}\left(c\right)$ 13: $b\leftarrow A_{2}^{\mathrm{Decaps}(msk,\cdot ),\mathrm{Compromise}(msk,\cdot ),\mathrm{PublicKey}(\cdot )}\left(st,\left(c,t\right),k_{kem}\right)$ 14: **return** *b*

The primary difference between Games 2 and 3 and Hybrid KEM Game 0 for $HKEM_{XtM}$ lies in the challenge. In Hybrid Game 2, we maintain consistency in the decapsulation query by checking if the ciphertext matches $c_{1}$, and if it does, we return the same random value every time. In Hybrid Game 3, this consistency is not maintained; each decapsulation of $c_{1}$ returns a freshly generated random key.

**Algorithm A2** Decapsulation Query for Hybrid Attack Games 2 and 3
1: Decaps(msk,id,c): 2: **if** Dict[id]≠∅ **then** 3: (pk,sk)←Dict[id] 4: **else** 5: $\left(pk,sk\right)\leftarrow \mathrm{HKEM}.\mathrm{LocalKeyGen}\left(1^{\lambda }\right)$ 6: Dict[id]←(pk,sk) 7: **end if** 8: $sk_{id}\leftarrow \mathrm{HKEM}.\mathrm{Extract}\left(msk,id\right)$ 9: $(c_{1}^{\prime },c_{2}^{\prime })\leftarrow c$ 10: $k_{1a}^{\prime }\|k_{1b}^{\prime }\leftarrow \mathrm{KEM}.\mathrm{Decaps}\left(sk,c_{1}^{\prime }\right)$ 11: **if** $c_{1}^{\prime }=c_{1}$ **then** ▹ Only in Hybrid Game 2 12: $k_{1a}^{\prime }\left\|k_{1b}^{\prime }\leftarrow k_{1a}\right\|k_{1b}$ ▹ Only in Hybrid Game 2 13: **end if** 14: $k_{2a}\|k_{2b}\leftarrow \mathrm{IDKEM}.\mathrm{Decaps}\left(sk_{id},c_{2}^{\prime }\right)$ 15: $k_{kem}\leftarrow k_{1a}^{\prime }⊕k_{2a}$ 16: $k_{mac}\leftarrow k_{1b}^{\prime }\|k_{2b}$ 17: **if** $t\neq {\mathrm{MAC}}_{k_{mac}}\left(c\right)$ **then** 18: **return** ⊥ 19: **else** 20: **return** $k_{kem}$ 21: **end if**

Clearly, distinguishing between Hybrid KEM Game 2 and Hybrid KEM Game 0 is as hard as distinguishing between KEM Game 0 and KEM Game 1: if an adversary B could distinguish between both of them, it could be used to produce an adversary $A_{1}$ capable of breaking the KEM security as described in Section 2.2. To show this, we build adversary $A_{1}$ as follows:

- Initialization: Adversary $A_{1}$ sets up the environment, initializing counters and variables, and selects one of the identities for the key encapsulation process (Algorithm A3).
  **Algorithm A3** Initialization for Adversary $A_{1}$
  1: **procedure** $A_{1}$($1^{\lambda },pk,c*,k*$) 2: Dict←EmptyDictionary() 3: $k\;\overset{\;\$}{\leftarrow }\;\left\{1,\ldots ,p\right\}$ 4: count←0 5: $\left(mpk,msk\right)\leftarrow HKEM.MasterKeyGen\left(1^{\lambda }\right)$ 6: B(mpk) ▹$A_{1}$ initializes adversary B by providing the mpk. 7: **end procedure**
- Decapsulation Query: Whenever B asks to decapsulate a ciphertext for a specific identity, $A_{1}$ either performs the decapsulation using the KEM or checks for the secret key to perform decapsulation for IDKEM:
  - If no key is found for the queried identity, a new key is generated and stored;
  - If the current query matches the chosen identity *k*, $A_{1}$ performs the decapsulation using its own decapsulation challenge from the KEM.
  Details are shown in Algorithm A4.
  **Algorithm A4** How Adversary $A_{1}$ responds to decapsulation queries.
  1: **procedure** $A_{1}$($msk,id_{i},c_{i}$) 2: $\left(pk_{i},sk_{i}\right)\leftarrow Dict.get\left(id_{i}\right)$ 3: **if** no key was found in Dict **then** 4: count←count+1 5: **if** count=k **then** 6: $Dict.store(id_{i},\left(pk,\emptyset \right))$ 7: $k_{i}\leftarrow Decaps\left(c_{i}\left[0\right]\right)$ ▹$A_{1}$ runs its own decapsulation query. 8: **else** 9: $\left(pk_{i},sk_{i}\right)\leftarrow HKEM.KeyGen\left(1^{\lambda }\right)$ 10: $Dict.store(id_{i},\left(pk_{i},sk_{i}\right))$ 11: **end if** 12: **end if** 13: Use either msk or the decapsulation of $c_{i}$ to compute the IDKEM decapsulation. 14: Provide a valid response to B. 15: **end procedure**
- Compromise Query: If B asks to compromise an identity, $A_{1}$ reveals the corresponding secret key unless the query matches the target identity *k*, in which case $A_{1}$ halts. If the compromised identity is the chosen target, $A_{1}$ halts because it cannot proceed with the game. Details are shown in Algorithm A5.
  **Algorithm A5** How Adversary $A_{1}$ responds to compromise queries.
  1: **procedure** $A_{1}$($id_{i}$) 2: **if** no key was found in Dict **then** 3: count←count+1 4: **if** count=k **then** 5: $A_{1}$ cannot succeed and halts. 6: **else** 7: $\left(pk_{i},sk_{i}\right)\leftarrow HKEM.KeyGen\left(1^{\lambda }\right)$ 8: $Dict.store(id_{i},\left(pk_{i},sk_{i}\right))$ 9: **end if** 10: **end if** 11: **if** $sk_{i}=\emptyset$ **then** 12: $A_{1}$ cannot succeed and halts. 13: **end if** 14: $sk_{id_{i}}\leftarrow HKEM.Extract\left(msk,id_{i}\right)$ 15: **return** $\left(sk_{i},sk_{id_{i}}\right)$ 16: **end procedure**
- Public Key Query: If B requests the public key of an identity, $A_{1}$ checks the dictionary and returns the corresponding public key or creates a new one. If the queried identity is the target *k*, $A_{1}$ returns the challenge’s public key pk. Details are shown in Algorithm A6.
  **Algorithm A6** How Adversary $A_{1}$ responds to public key queries.
  1: **procedure** $A_{1}$($id_{i}$) 2: $\left(pk_{i},sk_{i}\right)\leftarrow Dict.get\left(id_{i}\right)$ 3: **if** no key was found in Dict **then** 4: count←count+1 5: **if** count=k **then** 6: $Dict.store(id_{i},\left(pk,\emptyset \right))$ 7: **return** pk 8: **else** 9: $\left(pk_{i},sk_{i}\right)\leftarrow HKEM.KeyGen\left(1^{\lambda }\right)$ 10: $Dict.store(id_{i},\left(pk_{i},sk_{i}\right))$ 11: **end if** 12: **end if** 13: **return** $pk_{i}$ 14: **end procedure**
- Challenge: When B selects the target identity for its attack, $A_{1}$ obtains the identity public key from the dictionary. Then, it submits the challenge ciphertext c∗ and encapsulated key k∗ to B, which can be produced if the recovered public key is the same than the one that A received during initialization. Details are shown in Algorithm A7.
  **Algorithm A7** How Adversary $A_{1}$ responds when B chooses which identity it will attack.
  1: **procedure** $A_{1}$($id_{i}$) 2: $\left(pk_{i},sk_{i}\right)\leftarrow Dict.get\left(id_{i}\right)$ 3: **if** $pk_{i}\neq pk$ **then** 4: $A_{1}$ cannot succeed and halts. 5: **end if** 6: $A_{1}$ gets the challenge (c∗,k∗) from its challenger 7: **return** (c∗,k∗) 8: **end procedure**
- Finalization: When adversary B finalizes returning some bit *b*, adversary $A_{1}$ also finalizes sending this same value *b* to its own KEM challenger.

Note that when the adversary $A_{1}$ interacts with KEM Game 0, it produces responses for B that are consistent with Hybrid KEM Game 0. Similarly, when $A_{1}$ interacts with KEM Game 1, its responses are consistent with Hybrid KEM Game 2. The adversary $A_{1}$ produces a coherent output without halting only if it correctly guesses the value of *k* during initialization; otherwise, it halts during the challenge phase or in response to a decapsulation query. The probability of guessing correctly is 1/p. Assuming this, the probability of $A_{1}$ succeeding is exactly the same as the probability of B succeeding. Therefore, if $W_{0}$ denotes the event where adversary B outputs one in Hybrid KEM Game 0, and $W_{2}$ denotes the event in Hybrid KEM Game 2, we have the same probability of success for the corresponding adversary $A_{1}$:

$$Adv_{KEM}^{IND-CCA}\left(A_{1}\right)\geq \frac{1}{p}\left|Pr\left[W_{2}\right]-Pr\left[W_{0}\right]\right|$$

This result provides a negligible upper bound for distinguishing between the two games:

$$|Pr\left[W_{2}\right]-Pr\left[W_{0}\right]|\leq p\cdot Adv_{KEM}^{IND-CCA}\left(A_{1}\right)$$

Hybrid Game 3: This hybrid game is very similar to Hybrid Game 2. The key difference is that we no longer maintain consistent responses for decapsulation queries when the adversary attempts to decapsulate the value $c_{1}$. Instead, if such a query is made, we return the actual decapsulation result. In other words, we omit Lines 11 and 12 in the Decapsulation procedure described in Algorithm A2. Other than this change, Hybrid Game 3 is defined exactly as Hybrid Game 2. Notice that in Hybrid Game 3, the only way for the adversary to distinguish a difference in queries compared to Hybrid Game 2 is by making a decapsulation query where the second element matches the one in the challenged ciphertext, while the first element differs. Additionally, the adversary must produce a valid tag that is accepted by the MAC; otherwise, in both Hybrid games, the decapsulation query returns ⊥. Therefore, if there existed an adversary B capable of distinguishing between Hybrid Game 3 and Hybrid Game 2, we could construct an adversary $A_{2}$ that successfully wins the MAC Attack Game (Section 2.4). We build $A_{2}$ as follows:

- Initialization: Adversary $A_{2}$ aims to break the MAC security by controlling the rightmost bits of the key, which are generated by IDKEM. These bits cannot be treated as independent, random, and uniform in this game, so it is crucial to control them in the MAC key. The steps are as follows:
  1. We use the KEM, IDKEM, and MAC algorithms to initialize adversary B with key mpk, providing valid responses for all queries made by B. $A_{2}$ interacts with B in the same way as a legitimate challenger would, until B issues its challenge by selecting id as the target identity. We let pk be the corresponding public key associated with this identity;
  2. We use the IDKEM and KEM algorithms to generate an encapsulation $c^{*}$ for the identity id while also obtaining the secret $k_{2}$ produced by IDKEM and the encapsulation $c_{2}$;
  3. Return $\left(id,1,k_{2}\right)$
- Finalization: $A_{2}$ continues interacting with B to attempt to produce a forgery. It follows the steps below:
  1. Sends ((c∗,tag∗),k∗) as challenge to B. Here, c∗ is the encapsulation previously generated for id, tag∗ is the tag returned by the challenger, and k∗ is a random and uniformly chosen value;
  2. Continues using the KEM, IDKEM, and MAC algorithms to generate valid and consistent responses for all the queries made by B, provided that no decapsulation query involving $c_{1}$ is sent;
  3. If a decapsulation query involving $c_{1}$ and a given tag is received, it uses the Verification procedure to check the validity of the tag. If the tag is rejected, ⊥ is returned as response. Otherwise, $A_{2}$ halts and submits the tag as a successful forgery.

As discussed, the probability of $A_{2}$ succeeding is equivalent to the probability of B detecting a difference between Hybrid Game 2 and Hybrid Game 3, which corresponds to the probability that it forges a valid tag for the MAC. Therefore, we obtain the following upper bound:

$$|Pr\left[W_{3}\right]-Pr\left[W_{2}\right]|\leq Adv_{MAC}^{OT-sEUF}\left(A_{2}\right)$$

Finally, there is no difference between Hybrid Game 3 and KEM Game 1. In both games, the true decapsulation result is returned for all queries, without attempting to maintain consistency with the encapsulation from the challenge. In Hybrid Game 3, the secret is computed using a XOR operation between a random, uniform value and some other value $k_{2a}$ (as in Hybrid Attack Games 2 and 3, line 9). The result of this XOR operation is also a random and uniform value. Therefore, there is no difference between the two games:

$$|Pr\left[W_{1}\right]-Pr\left[W_{3}\right]|=0$$

By combining all these results, we conclude that for any adversary $A_{4}$, there exist adversaries $A_{1}$ and $A_{3}$ such that

$$Adv_{HKEM_{XtM}}^{IND-CCA}\left(A_{4}\right)\leq p\cdot Adv_{KEM}^{IND-CCA}\left(A_{1}\right)+Adv_{MAC}^{OT-sEUF}\left(A_{3}\right)$$

Therefore, if KEM is more secure than IDKEM, we can use this upper bound while ignoring the security of IDKEM. Step 2: Assuming IDKEM is more secure than KEM. In the previous step, we demonstrate that it is possible to ignore the security of IDKEM and state the security of HKEM purely in terms of the security of KEM and MAC. Thus, even if IDKEM is completely insecure, this does not necessarily compromise the security of HKEM. In this step, we show that it is also possible to express the security of HKEM solely in terms of IDKEM and MAC security, ignoring KEM. Therefore, even if KEM is completely insecure, HKEM still remains secure based on the other primitives. We now introduce the new hybrid games: Game $2^{\prime }$ and Game $3^{\prime }$. Since they are very similar to Games 2 and 3, we describe them briefly and explain how they help complete the proof. Hybrid Game $2^{\prime }$: The primary difference here is that instead of replacing the KEM secret (as in Line 8 of Hybrid Attack Games 2 and 3) with a random, uniform value, we replace IDKEM secret, i.e., “$k_{2a}\left|\right|k_{2b}\;\overset{\;\$}{\leftarrow }\;K$”. We then show that if an adversary can distinguish between Hybrid Game $2^{\prime }$ and Hybrid KEM Game 0, this adversary can be used to construct an adversary $A_{3}$ that breaks the IND-CCA security for IDKEM. The remainder of the proof is analogous, except that adversary $A_{3}$ does not need to guess which identity will be attacked. Since both KEMs are in the multi-user setting, $A_{3}$ can make queries to respond to all of B’s queries. Adversary $A_{3}$ chooses which identity to attack only after the adversary B makes its own choice. This provides us with the following upper bound:

$$|Pr\left[W_{2}^{\prime }\right]-Pr\left[W_{0}\right]|\leq Adv_{IDKEM}^{IND-CCA}\left(A_{3}\right)$$

Hybrid Game $3^{\prime }$: Similar to Hybrid Game 3, we modify the Decapsulation query in Hybrid Game $2^{\prime }$ to always return the correct decapsulation, without maintaining consistency with the random value that replaced the decapsulation of $c_{2}$ in Hybrid Game $2^{\prime }$. As before, distinguishing between Game $2^{\prime }$ and Game $3^{\prime }$ is possible only if an adversary can forge a valid MAC tag. In this case, $A_{3}$ works as before, but instead controlling the rightmost bits of the MAC key, it controls the leftmost bits which must be consistent with the secret $k_{1}$ produced by KEM. This modification does not affect the results; we obtain the same upper bound as before. By combining both cases, we conclude that for any adversary $A_{4}$, there exist adversaries $A_{1}$, $A_{2}$ and $A_{3}$ such that

$$Adv_{HKEM_{XtM}}^{IND-CCA}\left(A_{4}\right)\leq min\left\{p\cdot Adv_{KEM}^{IND-CCA}\left(A_{1}\right),Adv_{IDKEM}^{IND-CCA}\left(A_{2}\right)\right\}+Adv_{MAC}^{OT-sEUF}\left(A_{3}\right)$$

This upper bound concludes our security proof, demonstrating that under our assumptions, the advantage of any attacker in compromising the security of the scheme is negligible. □

## Appendix B

**Proof** **of Theorem 2.** As discussed in the text, we use a modified KEM Attack Game where the adversary attempts to distinguish between Game 0 and Game 1 or forge an ECDSA signature in either game. This augmented KEM Attack Game allows the adversary to query ECDSA signatures and assumes that the signature and the KEM (a DHKEM) share the same key. Following the proof in [26], the operation on Line 5 of the ECDSA signature algorithm (Section 2.5) is modeled as a composition of functions ψ∘Π∘φ that maps a group element to an integer modulo *q*. The function φ maps an elliptic curve point to a string encoding the x-coordinate of the point, Π is a bijective random oracle, and ψ performs the modulo *q* operation, where *q* is the elliptic curve group order. First, we create a Hybrid Attack Game 2 and demonstrate that if an adversary B can forge signatures or distinguish between Attack Game 0 and Game 1, then we can solve the computational Diffie–Hellman (CDH) problem using an adversary A. Adversary A does not precisely simulate Game 0 or Game 1 from the KEM Attack Game, but instead models a new Hybrid Game 2, which is a combination of the two. Adversary A operates as follows:

- Initialization: Adversary A is initialized with the security parameter $1^{\lambda }$ and a computational Diffie–Hellman instance $\left(G,g,q,y_{a}=g^{a},y_{b}=g^{b}\right)$, where its objective is to compute $g^{ab}$.
  To simulate the bijective random oracle Π:A→B, A initializes several lists:
  - $\Pi^{\to }$: stores tuples (a,b) with a∈A and b∈B for forward queries;
  - $\Pi^{\leftarrow }$: stores tuples (a,b,·) for backward queries;
  - DecapsList: stores values for consistent decapsulation responses;
  - OracleList: stores oracle query responses;
  - SignList: stores ECDSA signatures.
  Additionally, A samples *Q* distinct values $\beta_{1},\ldots ,\beta_{Q}$ from set *B* (expected number of queries to the random oracle) and initializes a counter t=0 to track queries.
  Finally, A initializes adversary B, providing it with randomness ρ, the security parameter $1^{\lambda }$, and the public key $pk=y_{a}$.
- Bijective Random Oracle Query: When adversary B queries $\Pi (a_{i})$, A first checks if $\left(a_{i},b_{i}\right)\in \Pi^{\to }$ or if $\left(a_{i},b_{i},\cdot \right)\in \Pi^{\leftarrow }$. If such a tuple exists, it returns $b_{i}$. Otherwise, it increments the counter *t*, stores $\left(a_{i},\beta_{t}\right)$ in $\Pi^{\to }$, and responds with $\beta_{t}$;
- Backward Bijective Random Oracle Query: When adversary B queries $\Pi^{-1}\left(b_{i}\right)$, A checks if $\left(a_{i},b_{i}\right)$ exists in either $\Pi^{\to }$ or $\Pi^{\leftarrow }$. If not, it selects a random $a_{i}\in A$, ensuring $a_{i}$ is not already in $\Pi^{\leftarrow }$ or $\Pi^{\to }$. If $a_{i}$ lies within the range of φ, A samples a random $\alpha \in Z_{q}$ and sets $a_{i}=\varphi \left(g^{\alpha }\right)$. It then stores $\left(a_{i},b_{i},\alpha \right)$ in $\Pi^{\leftarrow }$ and responds with $a_{i}$.
- KEM Random Oracle Query: When adversary B sends a query requesting the KEM random oracle output for message $m_{i}$, we proceed as follows:
  1. If $(m_{i},k_{i})\in OracleList$, $k_{i}$ is returned as a response;
  2. $m_{i}$ is attempted to parse as three elliptic curve points from the group *G*. If parsing fails, a random value $k_{i}$ is sampled uniformly from the oracle range, store $\left(m_{i},k_{i}\right)$ in OracleList and $k_{i}$ is returned as the response;
  3. $\left(y_{1},y_{2},y_{3}\right)$ are allowed to be the three elliptic curve points parsed from $m_{i}$. If $y_{3}\neq y_{a}$ or if $\left(y_{1},y_{2},y_{3}\right)$ is not a valid Diffie–Hellman tuple, a random value $k_{i}$ is sampled uniformly from the oracle range, $\left(m_{i},k_{i}\right)$ is stored in OracleList, and $k_{i}$ is returned as the response;
  4. If $\left(y_{1},y_{2},y_{3}\right)$ is a valid Diffie–Hellman tuple and $y_{3}=y_{a}$, it is determined whether $y_{2}=y_{b}$. If this holds, then $y_{1}=y^{ab}$, and a solutionis found to the computational Diffie–Hellman problem. If $y_{2}\neq y_{b}$, it is checked whether there is a tuple $\left(y_{2},k_{i}\right)$ in DecapsList. If such a tuple exists, $k_{i}$ is returned as the response. If no response is produced, a random value $k_{i}$ is sampled uniformly from the oracle range, $\left(m_{i},k_{i}\right)$ is stored in OracleList, and also $\left(y_{2},k_{i}\right)$ is stored in DecapsList to remember that $k_{i}$ is the correct decapsulation value for $y_{2}$ when using the public key $y_{a}$. Finally, $k_{i}$ is returned as the response.
- Signing Query: When adversary B sends a signing query with message $msg_{i}$, adversary A can simulate a valid signature, even without knowledge of the secret key, as follows. First, it selects a value $b_{i}$ uniformly at random from the bijective random oracle range *B*. If $\left(\cdot ,b_{i}\right)\in \Pi^{O}$ (which occurs with negligible probability), A is unable to produce a consistent signature and halts. Otherwise, A samples a random value $s_{i}\leftarrow Z_{q}$ and computes
  $$y^{\prime }\leftarrow g^{Hash\left(msg\right)s_{i}^{-1}}y_{a}^{\varphi \left(b_{i}\right)s_{i}^{-1}}$$
  Next, it computes $a_{i}\leftarrow \phi \left(y^{\prime }\right)$ and checks whether either $a_{i}$ or $b_{i}$ already appears in some tuple in $\Pi^{O}$. If either does, A fails to simulate a consistent bijective random oracle and halts. Otherwise, it stores $\left(a_{i},b_{i}\right)$ in $\Pi^{\leftarrow }$ and returns the signature $\left(\varphi \left(b_{i}\right),s_{i}\right)$ in response to the query.
  If an invalid value is generated when creating the signature (e.g., $r_{i}=\varphi \left(b_{i}\right)=0$ or $s_{i}=0$), A can attempt the process again, as in standard signature generation. All messages and signatures generated through this query are stored in the list SignList;
- Challenge: To generate the challenge, adversary A constructs the KEM challenge by selecting $y_{b}=g^{b}$ as the ciphertext *c*. The correct secret should be the output of the random oracle for the input $g^{ab}\left|g^{b}\right|g^{a}$. However, since A does not know the value of $g^{ab}$, it instead selects a value *k* uniformly at random from the oracle range and sends $\left(g^{b},k\right)$ as the challenge.
  Since the secret *k* is chosen randomly and independently of the ciphertext, this behavior is indistinguishable from that of Game 1, where the secret is also chosen at random.
- Decapsulation Query: When adversary B queries the decapsulation of some ciphertext $c_{i}\neq y_{b}$, adversary A checks if a corresponding tuple $\left(c_{i},k_{i}\right)$ exists in the DecapsList. If such a tuple is found, it returns the value $k_{i}$ as the response. Otherwise, A selects a value k uniformly at random from the range of the random oracle, stores $\left(c_{i},k_{i}\right)$ in the DecapsList, and returns $k_{i}$ as the response.
- Finalization: After all queries are processed, we assume that adversary B produces a bit as output, attempting to distinguish whether it was in Game 0 or Game 1, as well as a potential forgery for the ECDSA signature.

As mentioned earlier, the game that adversary A simulates is indistinguishable from Game 1 because the secret *k* sent during the challenge was chosen uniformly at random and independently of the ciphertext $c=g^{b}$. However, given the nature of the random oracle, the only way to determine that the secret *k* is random and not the decapsulation of $g^{b}$ is to identify that the oracle output for $g^{ab}∥g^{b}∥g^{a}$ (the real encapsulation of $g^{b}$) is different from *k*. Adversary A only discovers this if it queried the oracle with $g^{ab}∥g^{b}∥g^{a}$. In doing so, it reveals the solution $g^{ab}$ to the computational Diffie–Hellman (CDH) problem. Thus, even when using the keys to generate signatures, breaking the DH-KEM is as hard as solving the CDH problem. Now, we suppose a valid forgery $\left(msg^{*},\left(r^{*},s^{*}\right)\right)$ is produced for the ECDSA signature. From these values, adversary A can compute

$$y^{*}\leftarrow g^{Hash\left(msg^{*}\right)/s^{*}}y_{a}^{r^{*}/s^{*}}.$$

We let *k* be the discrete logarithm of $y^{*}$. We know from the exponents that

$$ks^{*}=Hash\left(msg^{*}\right)+ar^{*},$$

even without knowledge of the values *k* and *a*. At this point, it is possible to compute $a^{*}\leftarrow \varphi \left(y^{*}\right)$, and we can assume, without loss of generality, that a pair in $\Pi^{\to }$ or $\Pi^{\leftarrow }$ contains $a^{*}$ as the first element. This could occur either because adversary A queried it from the bijective random oracle or because this value was produced during a signing query. Case 1: if $\left(a^{*},b^{*}\right)\in \Pi^{\to }$ was produced during a signing query, A must have also generated a past signature in the form $\left(msg^{\prime },\left(r^{\prime },s^{\prime }\right)\right)$ such that

$$y^{\prime }=g^{Hash\left(msg^{\prime }\right)/s^{\prime }}y_{a}^{r^{\prime }/s^{\prime }}\;\mathrm{and}\;y^{\prime }=g^{\pm k*}.$$

By searching in SignList, adversary A can recover these values. This yields the following equation:

$$Hash\left(msg^{*}\right)/s^{*}+ar^{*}/s^{*}=\pm \left(Hash\left(msg^{\prime }\right)/s^{\prime -1}+ar^{\prime }/s^{\prime }\right),$$

from which adversary A can compute *a* and and subsequently derive $y_{b}^{a}=g^{ab}$, solving the computational Diffie–Hellman problem. Case 2: If $\left(a^{*},b^{*},\alpha \right)\in \Pi^{\leftarrow }$, the tuple was produced by a backward bijective random oracle query. In this case, we know the value α such that $g^{\pm \alpha }=y^{*}$. From this, and the definition of $y^{*}$, adversary B obtains the following equation:

$$\pm \alpha =Hash\left(msg^{*}\right)/s^{*}+ar^{*}/s^{*},$$

which allows it to isolate the discrete logarithm *a* and solve the computational Diffie–Hellman problem. Case 3: If $\left(a^{*},b^{*}\right)\in \Pi^{\to }$ was produced by a bijective random oracle query, then $b^{*}=\beta_{j}$ for some 0<j≤Q. In this case, adversary A can generate a new sequence of distinct values from $B:\beta_{j}^{\prime },\beta_{j+1}^{\prime },\ldots ,\beta_{Q}^{\prime }$, and run adversary B again using fresh lists but the same randomness source ρ and input as before. The only difference is that when the counter *t* increments to some value I≥j, A uses the new value $\beta_{i}^{\prime }$ instead of the previous one. By the Forking Lemma, if A produces a forgery of this type with non-negligible probability, then with non-negligible probability, two forgeries can be obtained from both executions: one with the value $\beta_{j}$ in the signature and the other with $\beta_{j}^{\prime }$. If not, A fails and aborts. We let $\left(msg^{**},\left(r^{**},s^{**}\right)\right)$ be the second forgery. Combining the signatures from both executions, adversary A can derive the following equation:

$$\pm \left(Hash\left(msg^{*}\right)/s^{*}+ar^{*}/s^{*}\right)=Hash\left(msg^{**}\right)/s^{**}+ar^{**}/s^{**},$$

from which it can compute the discrete logarithm *a*, thereby solving the computational Diffie–Hellman problem. The two additional possibilities not previously discussed are:

1. Adversary B produces a forgery by finding a collision in the hash function;
2. In the final equation, an adversary could produce values such that $hash\left(msg^{**}\right)/r^{**}=Hash\left(msg^{*}\right)/r^{*}$.

In either of these cases, adversary A would fail to compute the discrete logarithm. However, these events occur with negligible probability if *G* is collision-resistant and has high division entropy resistance, as required for the security of the ECDSA signature scheme (as discussed in [26]). Thus, except with negligible probability, adversary A successfully simulates a bijective random oracle (we omit the calculation of these probabilities since they are identical to those found in the ECDSA proof in [26]). If adversary B forges a signature, adversary A can compute the solution to the computational Diffie–Hellman problem. Furthermore, adversary A can also compute a solution with non-negligible probability if adversary B breaks the IND-CCA2 security of the DH-KEM with non-negligible probability. □
